# Ethanol and unsaturated dietary fat induce unique patterns of hepatic ω-6 and ω-3 PUFA oxylipins in a mouse model of alcoholic liver disease

**DOI:** 10.1371/journal.pone.0204119

**Published:** 2018-09-26

**Authors:** Dennis R. Warner, Huilin Liu, Shubha Ghosh Dastidar, Jeffrey B. Warner, Md Aminul Islam Prodhan, Xinmin Yin, Xiang Zhang, Ariel E. Feldstein, Bin Gao, Russell A. Prough, Craig J. McClain, Irina A. Kirpich

**Affiliations:** 1 Division of Gastroenterology, Hepatology, and Nutrition, Department of Medicine, University of Louisville, Louisville, Kentucky, United States of America; 2 College of Life Science, Jilin University, Changchun, China; 3 Department of Pharmacology and Toxicology, University of Louisville School of Medicine, Louisville, Kentucky, United States of America; 4 Department of Chemistry, University of Louisville, Louisville, Kentucky, United States of America; 5 University of Louisville Alcohol Center, University of Louisville School of Medicine, Louisville, Kentucky, United States of America; 6 Hepatobiology & Toxicology Program, University of Louisville, Louisville, Kentucky, United States of America; 7 Division of Gastroenterology, Department of Pediatrics, University of California San Diego, San Diego, California, United States of America; 8 Laboratory of Liver Diseases, National Institute on Alcohol Abuse and Alcoholism, National Institutes of Health, Bethesda, Maryland, United States of America; 9 Robley Rex Veterans Medical Center, Louisville, Kentucky, United States of America; INRA, FRANCE

## Abstract

Alcoholic liver disease (ALD), a significant health problem, progresses through the course of several pathologies including steatosis, steatohepatitis, fibrosis, and cirrhosis. There are no effective FDA-approved medications to prevent or treat any stages of ALD, and the mechanisms involved in ALD pathogenesis are not well understood. Bioactive lipid metabolites play a crucial role in numerous pathological conditions, as well as in the induction and resolution of inflammation. Herein, a hepatic lipidomic analysis was performed on a mouse model of ALD with the objective of identifying novel metabolic pathways and lipid mediators associated with alcoholic steatohepatitis, which might be potential novel biomarkers and therapeutic targets for the disease. We found that ethanol and dietary unsaturated, but not saturated, fat caused elevated plasma ALT levels, hepatic steatosis and inflammation. These pathologies were associated with increased levels of bioactive lipid metabolites generally involved in pro-inflammatory responses, including 13-hydroxy-octadecadienoic acid, 9,10- and 12,13-dihydroxy-octadecenoic acids, 5-, 8-, 9-, 11-, 15-hydroxy-eicosatetraenoic acids, and 8,9- and 11,12-dihydroxy-eicosatrienoic acids, in parallel with an increase in pro-resolving mediators, such as lipoxin A4, 18-hydroxy-eicosapentaenoic acid, and 10S,17S-dihydroxy-docosahexaenoic acid. Elucidation of alterations in these lipid metabolites may shed new light into the molecular mechanisms underlying ALD development/progression, and be potential novel therapeutic targets.

## Introduction

Alcoholic liver disease (ALD) is a significant human health problem with global ramifications due to its significant morbidity, mortality, and burden on the health care system [1]. The clinical manifestations of ALD includes a wide spectrum of pathological conditions ranging from hepatic steatosis, an accumulation of lipid droplets within hepatocytes, to alcoholic steatohepatitis, in which hepatic lipid deposition is coupled with marked inflammation. Alcoholic steatohepatitis can further progress to hepatic fibrosis, cirrhosis, and in some cases, hepatocellular carcinoma. ALD pathogenesis is multifactorial, where an interplay between ethanol (EtOH) consumption and genetic, epigenetic and environmental factors (*e*.*g*., diet) contribute to liver damage. There are no effective FDA-approved medications to prevent or treat any stages of ALD, and the molecular mechanisms underlying the development and progression of EtOH-induced liver pathology are not completely understood. Thus, the identification of new mechanisms and novel therapeutic targets in ALD is of paramount importance.

Alterations in polyunsaturated fatty acid (PUFA)-derived lipid metabolites have been linked to the pathogenesis of numerous diseases, including ALD and nonalcoholic fatty liver disease (NAFLD) [2–8]. The severity of experimental ALD in a rat model was positively correlated with the hepatic arachidonic acid (AA) metabolites, leukotriene B4 and thromboxane B2 [9]; and the inhibition of the thromboxane production or thromboxane receptor function, attenuated EtOH-induced inflammatory and fibrotic changes [10]. Increased plasma levels of 5-, 12-, and 15 HETEs (oxidized metabolites of AA), and 9- and 13- hydroxy-octadecadienoic acids (HODEs, oxidized metabolites of linoleic acid, [LA]) were found in plasma of patients with alcoholic cirrhosis [3]. Oxidative PUFA metabolites (oxylipins) are diverse bioactive lipid mediators that are synthesized from ω-3 and ω-6 PUFAs, and exert pro-inflammatory or dual anti-inflammatory and pro-resolution of inflammation activity [11, 12]. In mammals, oxylipins are primarily formed through four major routes: 1) cyclooxygenases (COXs), 2) lipoxygenases (LOXs), 3) cytochrome-P450 epoxygenases (CYPs), and 4) non-enzymatic autoxidation, particularly during times of oxidative stress. In some instances, the same metabolite can be produced from more than one pathway. Many of these compounds can be further metabolized via multiple routes; for example, hydrolysis of epoxides (epoxy-FAs, CYP metabolites) to their corresponding dihydroxy-FAs through the action of soluble epoxide hydrolase (sEH) [13]. The biological effects of distinct metabolites depend on the target tissue, concentrations, presence of other mediators, receptors, the nature of downstream signaling, and other factors.

Studies from our laboratory and others have demonstrated that both alcohol and specific dietary fats play important roles in the pathogenesis of ALD [14]. Accumulating evidence using rodent models of ALD has revealed the deleterious effects of ω-6 PUFAs, specifically LA, which may be partially attributed to the increased levels of pro-inflammatory oxidized LA metabolites derived via the lipoxygenase (LOX) pathway [15, 16]. However, less is known of the role of oxidized PUFA metabolites generated through other metabolic pathways (*e*.*g*., CYP/sEH), as well as lipid mediators derived from ω-3 PUFAs, such as α-linolenic acid (ALA), eicosapentaenoic acid (EPA) and docosahexaenoic acid (DHA). Given that the majority of PUFA metabolites are potent endogenous signaling molecules that function through multiple pathways, identification of changes in specific lipid mediators might shed new light into the mechanisms contributing to ALD pathogenesis, and may reveal novel therapeutic targets and biomarkers of this disease. Because there are no FDA-approved therapies for ALD, the potential benefits of oxylipins derived from ω-3 PUFAs (*e*.*g*., resolvins, protectins, and maresins) [17], and specific PUFA-CYP-derived metabolites (PUFA epoxides) [18] make these molecules potential candidates as new therapies for ALD.

The goal of the present study was to identify changes in oxylipin profiles (bioactive, oxidized metabolites of ω-3 and ω-6 PUFAs) in the livers of mice fed ethanol and a diet high in saturated fat (SF) or unsaturated fat (USF). Identification of these changes may provide a better understanding of the roles that specific dietary fats have in promoting or preventing ALD and lead to development of therapies targeting this unique class of PUFA metabolites.

## Materials and methods

### Animals and treatments

**Fig 1:**
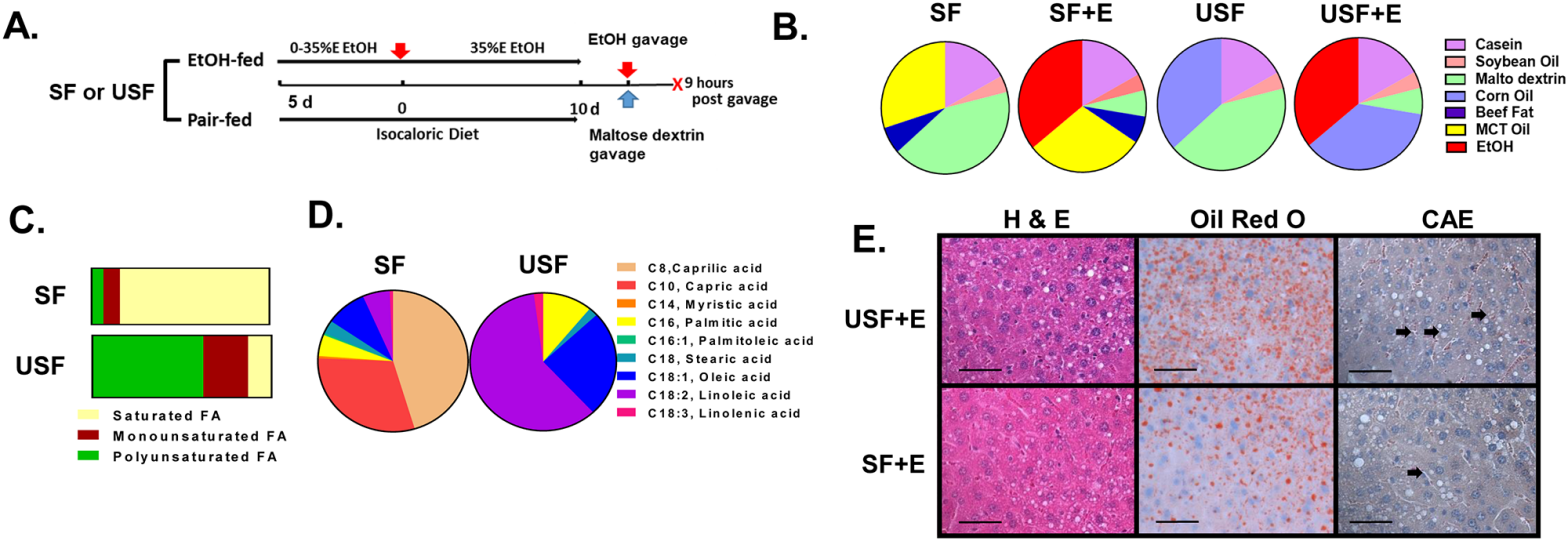
Characterization of liver injury caused by chronic-binge ethanol exposure. A: Schematic presentation of the chronic-binge ethanol exposure protocol. C57BL/J mice were fed control (pair-fed) or ethanol diets for 10 days, followed by single gavage of maltose dextrose or ethanol on day 11, respectively. Animals were euthanized 9 hours after gavage. B: The composition of the experimental liquid diets. The SF diet was enriched in beef tallow fat and MCTs (18:82 ratio). The USF diet was enriched with corn oil. Soybean oil was used in both diets to provide essential free fatty acids. The control (SF and USF) diets contained 43% of calories from carbohydrate, 17% from protein, and 40% from fat. The SF and ethanol and USF and ethanol diets contained 35% of calories from ethanol to replace the calories from carbohydrate. C: The percent of saturated, monounsaturated and polyunsaturated fatty acids was 84.1%, 9.9%, and 6.6% in SF diet and, 13.1%, 25.0%, and 61.9% in USF diet, respectively. D: Fatty acid composition of experimental diets. E: Representative images of hepatic hematoxylin and eosin (H&E), Oil Red O and chloroacetate esterase (CAE) staining. Original magnification, x400, scale bars are 50 μm. Arrows indicate CAE-positive neutrophils.

Male mice (C57BL/6, 8 weeks old) were purchased from Jackson Lab (Bar Harbor, ME) and housed in a specific pathogen-free animal facility accredited by the Association for Assessment and Accreditation of Laboratory Animal Care. A chronic-binge experimental animal model of ALD, which mimics the pathology of human AH, was utilized in this study [19]. Briefly, mice were fed control or EtOH-containing (5% EtOH w/v) diets *ad libitum* for 10 days followed by a single binge of EtOH delivered by oral gavage (5 g/kg of a 20% v/v ethanol solution) on day 11, whereas mice in control groups were gavaged with an isocaloric/isovolumetric maltodextrin solution. The animals were euthanized 9 hours later. A schematic diagram of the experimental design is shown in Fig 1A. The mice were fed Lieber–DeCarli liquid diets (Research Diet, New Brunswick, NJ) containing either saturated fat (SF, enriched in beef tallow:medium chain triglycerides [MCT], 18:82 ratio) or unsaturated fat (USF, enriched in corn oil). In pair-fed controls, the levels of protein, carbohydrate, and fat were held constant at 17, 43, and 40% of total caloric energy, respectively. In the EtOH-containing diets, EtOH (35% of total calories) was substituted for carbohydrate energy (Fig 1B). The percent of saturated, monounsaturated and polyunsaturated fatty acids was 84.1%, 9.9%, and 6.6% in the SF diet and, 13.1%, 25.0%, and 61.9% in the USF diet, respectively (Fig 1C). Octanoic (caprylic, C_8_H_16_O_2_) and decanoic (capric, C_10_H_20_O_2_) were the major fatty acids (FAs) in the SF diet, whereas octadecadienoic (linoleic, LA-ω6, C_18_H_32_O_2_) and octadecenoic (oleic, C_18_H_34_O_2_) were the major FAs in the USF diet (Fig 1D). Food was prepared freshly each day and consumption was monitored daily. The control groups (the pair-fed mice) received the same amount of isocaloric food (maltodextrin containing diets) that ethanol-fed animals consumed in the previous day (pair-feeding protocol). At the conclusion of the experiment, mice were euthanized and blood was collected from the inferior vena cava via venipuncture using heparinized syringes. Plasma was separated by centrifugation, aliquoted, and stored at -80°C for further analysis. Portions of liver tissue from the left hepatic lobe were snap-frozen in liquid nitrogen and stored at -80°C or were fixed in 10% neutral buffered formalin and embedded in paraffin for further analysis. The study protocol (number 12048) was approved by the University of Louisville Institutional Animal Care and Use Committee.

### Liver histological analysis and staining

Sections (5 μm) were obtained from formalin-fixed, paraffin-embedded liver tissue, and stained with hematoxylin and eosin for histological evaluation of liver injury. Slides were evaluated by an investigator who was blinded as to the treatment groups. Hepatic lipid content was determined following Oil-Red-O staining of optimal cutting temperature compound (OCT, Sikura Finetech, Torrance, CA)-embedded samples. Briefly, cryosections were dried and then fixed in 60% (v/v) isopropanol for 10 min, stained for 30 min with a 0.3% Oil-Red-O solution prepared in 60% isopropanol, and then washed three times with 60% isopropanol. Hepatic neutrophil infiltration was assessed by chloroacetate esterase (CAE) staining using a commercially available kit (Sigma, St. Louis, MO). Quantification of CAE staining was performed by counting CAE-positive neutrophils in a random series of 5 digital images per tissue section. The number of CAE-positive cells was then summed and averaged to obtain an estimate for each mouse (n = 6–8 animals/group).

### Assessment of liver triglycerides

Hepatic lipids were extracted with chloroform and methanol as previously described [20], and triglycerides (TGs) were measured using reagents from Thermo Fisher Scientific Inc. (Waltham, MA) according to the manufacturers’ instructions.

### Blood biochemical analysis

Plasma levels of alanine aminotransferase (ALT, a marker of liver injury) were determined using the Piccolo Xpress analyzer (Abaxis, Union City, CA) according to the manufacturer’s instructions.

### RNA isolation and real time quantitative reverse transcription PCR (qRT-PCR)

RNA was purified with Trizol reagent (Thermo Fisher) as described by the manufacturer, and digested with DNase I (Thermo Fisher). cDNA was synthesized with qScript cDNA Supermix (Quanta Biosciences, Beverly, MA) and a cDNA equivalent of 10 ng RNA (or 0.1 for 18S ribosomal RNA) was analyzed in each qPCR reaction. RT—PCR assays were performed with PerfeCTa SYBR Green Fast Mix (Quanta Biosciences, Beverly, MA) on the Applied Biosystems 7900HT platform (Foster City, CA). Primer sequences are listed in S1 Table.

### Western blotting

Nuclear and cytoplasmic fractions of liver tissue were prepared using the NE-PER kit (Thermo Fisher) supplemented with protease and phosphatase inhibitors (Sigma, St. Louis, MO) and protein concentrations were determined using the Bradford Protein Assay reagent (Bio-Rad, Hercules, CA). Fifty-60 μg was separated by SDS-PAGE and transferred to PVDF membranes. Blots were probed using the antibodies listed in S2 Table and data was quantified using Image J [21].

### Hepatic free fatty acid analysis

Hepatic free FA analysis was performed in the Center for Regulatory and Environmental Analytical Metabolomics at the University of Louisville by comprehensive two-dimensional gas chromatography mass spectrometry as previously described [22, 23] (see details in S1 Methods).

### Hepatic PUFA metabolite analysis

Lipid extraction from liver tissue and quantification of lipid metabolite levels were performed as previously described [24–27] by the Wayne State University Lipidomic Core Facility (supported by NIH grant S10RR027926). Briefly, tissue samples were homogenized in phosphate buffer, pH 7.2. Six samples from each group were spiked with internal standards and extracted for PUFA metabolites on C18 columns as described earlier [24–27]. High-performance liquid chromatography was performed on a Prominence XR system (Shimadzu Corp., Kyoto, Japan) using a Luna C18 (3μ, 2.1x150 mm) column. Mass spectra for each detected lipid mediator were recorded using the Enhanced Product Ion feature to verify the identity of the detected peak in addition to MRM transition and retention time match with the standard. The data were collected using Analyst 1.6.2 software and the MRM transition chromatograms were quantitated by MultiQuant software (both from SCIEX, Framingham, MA). The internal standard signals in each chromatogram were used to normalize instrument signals that were further used for recovery as well as for relative quantitation of each analyte.

### Statistics and data analysis

Results are expressed as mean ± standard error of the mean (SEM). Statistical significance was determined using Two-Way Analysis of Variance (ANOVA). A *p*-value of < 0.05 was considered statistically significant. The correlation between various characteristics was assessed using Pearson correlation analysis. Statistical analysis was performed using GraphPad Prism 5.01 software (GraphPad Software, Inc., La Jolla, CA). The analytical platform used for the hepatic PUFA metabolite analysis can detect 139 individual lipid metabolites. Of these, 79 metabolites, were detected in at least 4 samples in each of all 4 groups (SF, USF, SF+EtOH, USF+EtOH), and those 79 metabolites were included into data analysis. A similar approach was used for the analysis of hepatic fatty acid abundance.

## Results

### Characterization of liver injury caused by chronic-binge ethanol exposure with USF or SF diet

The animal model used in the current study (chronic-binge EtOH exposure) was developed with an EtOH-containing liquid diet enriched with USF and recapitulates features of human alcoholic hepatitis, including liver inflammation and elevated neutrophil infiltration [19]. Here, we examined the effect of USF versus SF on chronic-binge EtOH-induced liver damage. As shown in Fig 1E and Table 1, chronic-binge EtOH exposure coupled with an USF diet, when compared to a SF diet, resulted in greater liver damage displaying early stage of ALD characterized by hepatic steatosis (confirmed by histological assessment of liver sections, Oil Red O staining, and increased hepatic TGs), and liver injury with elevated plasma ALT levels. Although there was an increase in plasma ALT levels in the SF+EtOH group when compared to the control group, it did not reach statistical significance. Although hepatic TGs were elevated in both the SF-EtOH and USF-EtOH-fed groups compared to pair-fed control animals, this increase was more pronounced in mice fed USF+EtOH. Moreover, we observed greater up-regulation of hepatic pro-inflammatory genes, including tumor necrosis factor alpha (*Tnf-α)*, interleukin-1 beta (*Il-1β)*, and monocyte chemotactic protein-1 (*Mcp1)*, and increased neutrophil infiltration in the USF+EtOH group when compared to USF controls or SF+EtOH-fed animals. These data demonstrate that USF caused greater liver damage than SF in this chronic-binge EtOH feeding model.

**Table 1:** Characteristics of liver injury caused by chronic-binge ethanol exposure and dietary USF or SF.

Parameters	SF	SF+EtOH	USF	USF+EtOH	Two-Way ANOVA, *P* values		
					*P*_*1*_	*P*_*2*_	*P*_*3*_
**Blood Characteristics**							
ALT, U/L	41.78±4.93	93.38±10.54	47.50±6.66	194.6+53.51 ^a^^b^	0.0004	0.0429	0.0683
TGs, mg/dl	39.20±1.91	48.00±5.00	40.20±1.21	46.43+4.01	0.9271	0.0213	0.6809
**Liver Parameters**							
TGs, mg/g liver	10.38±4.90	25.87±2.88 ^d^	20.15±2.97	38.17±4.14 ^a^^b^	0.0003	0.0132	0.6554
*Hmgb1* mRNA	1.01±0.05	1.05±0.06	0.79±0.04	1.21±0.09 ^b^	0.0098	0.7157	0.0467
*Xbp1* mRNA	1.25±0.26	1.08±0.27	0.60±0.19	1.66±0.20 ^b^	0.1482	0.9026	0.0490
*Tnf- α* mRNA	0.95±0.09	1.12±0.13	0.93±0.08	1.66±0.20 ^a^^b^	0.0009	0.0495	0.0353
*Mcp1* mRNA	1.12±0.20	2.11±1.3	1.50±0.21	3.11±0.93 ^b^	0.0112	0.1624	0.5232
*Il-1β* mRNA	1.06±0.07	1.12±0.10	1.06±0.08	1.62±0.26 ^a^^b^	0.0295	0.0773	0.0767

Data are presented as the mean ± SEM, n = 9–10 animals per group. Two-way ANOVA was performed to assess the contribution of the ethanol, diet, and their interactions. *P*_*1*_ is the *P* value of ethanol factor, *P*_*2*_ is the *P* value of a diet factor, *P*_*3*_ is the *P* value of the interaction between the diet and ethanol. Values with different superscripts differ significantly (*P* < 0.05).

^a^ SF+EtOH vs USF+EtOH;

^b^ USF+EtOH vs USF;

^c^ SF vs USF;

^d^ SF vs SF+EtOH.

mRNA expression was measured by real-time PCR, and is expressed as fold- change relative to the SF pair-fed group. ALT, alanine aminotransferase; Hmgb1, High mobility group box 1 protein; Il-1β, interleukin-1 beta; Mcp1, monocyte chemoattractant protein 1; SF, saturated fat; TGs, triglycerides; Tnf-α, tumor necrosis factor alpha; USF, unsaturated fat; Xbp1, X-box binding protein 1.

**Fig 2:**
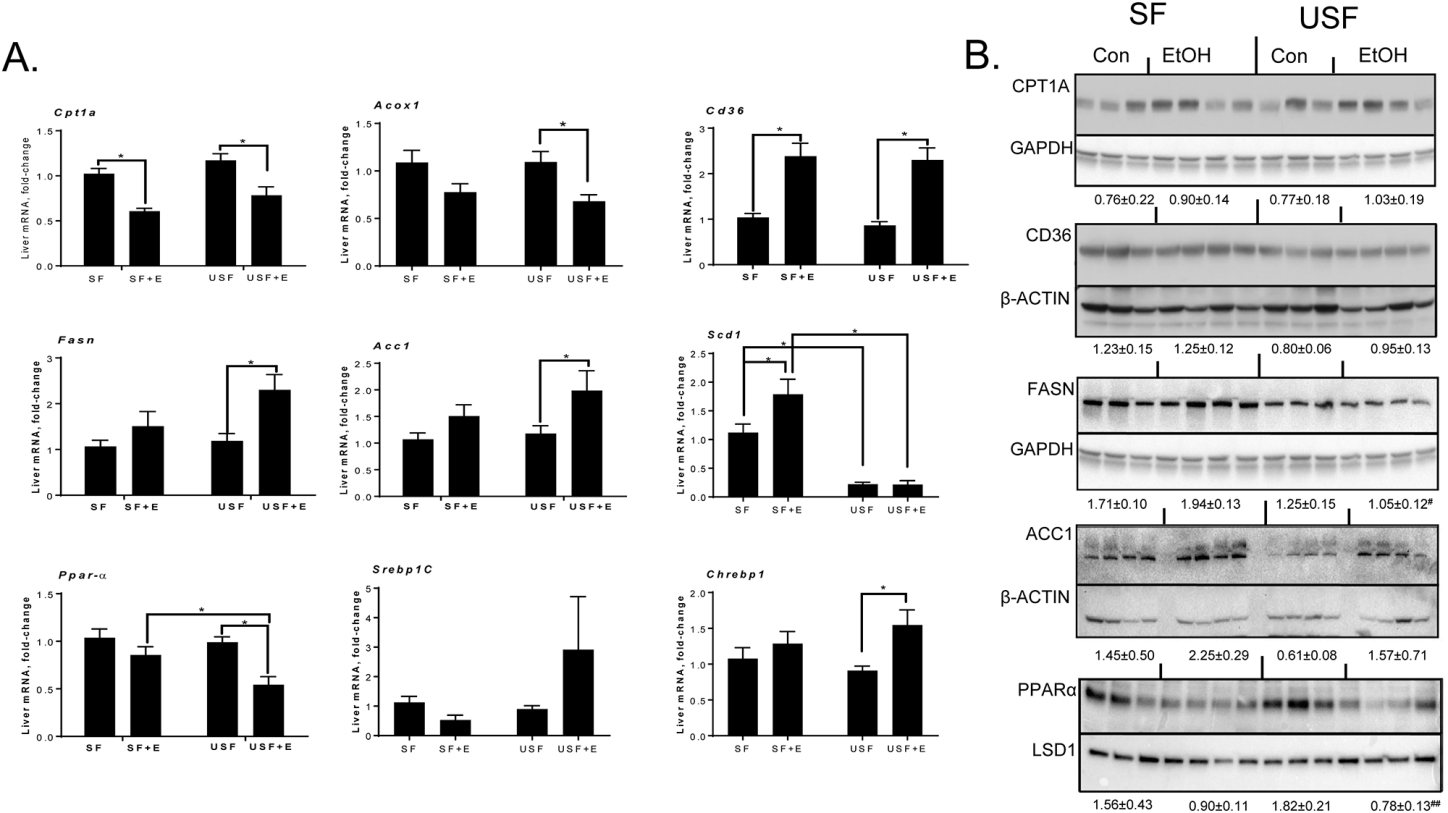
Alterations in the regulation of lipid metabolism in mice fed SF or USF following EtOH administration. Gene (panel A) and protein (panel B) expression of key enzymes and transcription factors involved in the regulation of lipid homeostasis. qPCR data are expressed as the fold-change relative to the SF pair-fed group (mean ± SEM, n = 8–10). Western blots were quantified using Image J and normalized to the respective loading controls and presented below each group (mean ± SEM, n = 3–4). * *P* < 0.05, two-way ANOVA; ^#^ *P*<0.05 vs. control; ^##^ *P*<0.05 vs. SF+EtOH; E, ethanol; SF, saturated fat; USF, unsaturated fat.

To identify potential mechanisms contributing to chronic-binge EtOH-induced liver steatosis, the expression of key genes involved in lipid metabolism was evaluated (Fig 2). In comparison to pair-fed mice, expression of carnitine palmitoyltransferase 1A gene (*Cpt1a*, a rate-limiting enzyme in fatty acid β-oxidation) was significantly decreased in response to EtOH consumption in both the SF and USF groups. However, the differences in CPT1A protein levels were not observed in these mice. Another gene involved in β-oxidation, Acyl-CoA Oxidase 1 (*Acox1)*, was also decreased by EtOH in both diet groups, reaching statistical significance in the USF+EtOH group. Expression of fatty acid translocase/cluster of differentiation 36 mRNA (*Fat/Cd36*, a key transporter involved in fatty acid uptake across the plasma membrane) was similarly elevated in response to ethanol exposure in the livers of both SF and USF fed mice compared to control pair-fed animals, while FAT/CD36 protein levels were constant. Expression of fatty acid synthase (*Fasn*) and acetyl-CoA carboxylase 1 (*Acc1*), encoding key rate-limiting enzymes involved in fatty acid biosynthesis, were both up-regulated in the livers of mice fed USF+EtOH compared to pair-fed control animals, whereas the expression of these genes was unaltered in SF+EtOH-fed mice. While, FASN protein levels were similar in both the USF+EtOH and pair-fed control mice, increased ACC1 protein levels in EtOH-fed mice were in agreement with mRNA expression. The expression of stearoyl-CoA desaturase-1 (*Scd1*), which catalyzes the rate-limiting step in monounsaturated fatty acid (MUFA) synthesis from SFs, was increased by EtOH in the SF diet group. Low levels of *Scd1* were observed in the USF compared to SF diet-fed animals. Among transcriptional factors involved in the regulation of lipid metabolism, the expression of hepatic peroxisome proliferator-activated receptor α (*Ppar-α*, a key transcriptional factor regulating fatty acid β-oxidation) was significantly decreased in the livers of USF+EtOH-fed mice compared to controls and SF+EtOH-fed animals. At the protein level, PPARα was significantly decreased by EtOH in the USF diet group. Transcription factors regulating *de novo* lipogenesis, such as the sterol regulatory element-binding protein 1-c (*Srebp1c*) and the carbohydrate response element binding protein, *Chrebp*, were elevated predominantly in USF+EtOH fed mice compared to pair-fed controls.

### Hepatic fatty acid composition in mice exposed to chronic-binge ethanol administration

**Fig 3:**
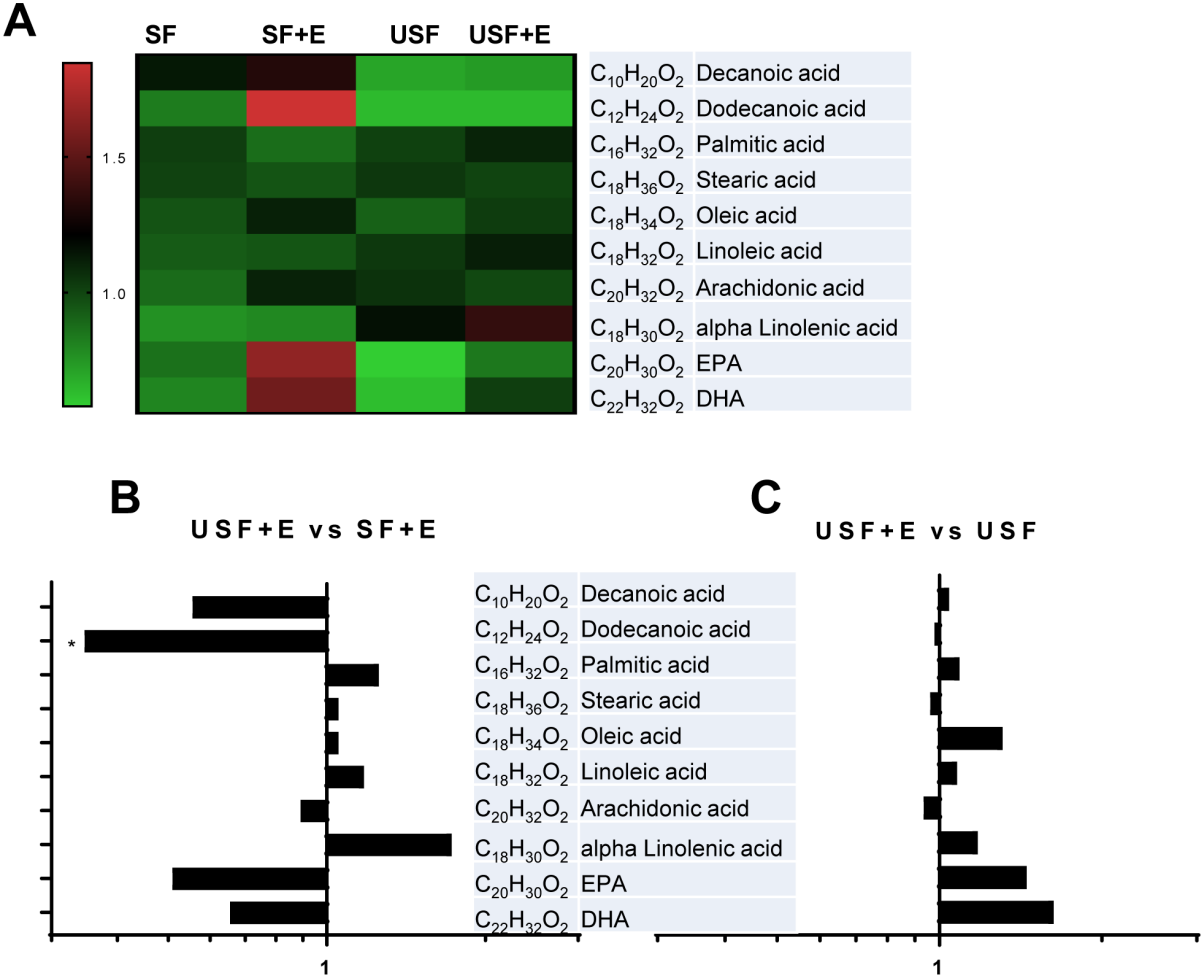
Hepatic fatty acid composition in mice exposed to chronic-binge ethanol administration. A: Changes in the abundance of individual hepatic fatty acids between experimental groups. Results are expressed as a matrix view (heat map) where rows represent individual fatty acid and columns represent group distribution. The intensity of each color denotes the standardized ratio between each sample value and the average levels of each individual fatty acid across all samples. B: Comparison of hepatic fatty acids between USF+E and SF+E. C: Comparison of hepatic fatty acids between USF+E and USF groups. Data are expressed as a fold changes. * *P* < 0.05. DHA, docosahexaenoic acid; E, ethanol; EPA, eicosapentaenoic acid; SF, saturated fat; USF, unsaturated fat.

We then performed an analysis of hepatic FAs to identify specific changes induced by EtOH that may contribute to the liver pathology and may modulate the profile and levels of FA-derived metabolites (Fig 3 and S3 Table). As expected, the levels of medium chain FAs, including decanoic (capric, C_10_H_20_O_2_), and dodecanoic (lauric, C_12_H_24_O_2_) acids were higher in mice fed SF and SF+EtOH compared to animals fed USF or USF+EtOH (Fig 3A). There were no significant changes in the content of long chain FAs, including the saturated FAs, palmitic (C_16_H_32_O_2_) and stearic (C_18_H_36_O_2_) acids, the monounsaturated FA oleic acid (C_18_H_34_O_2_, ω9), or the ω-6 PUFA, arachidonic acid (AA, C_20_H_32_O_2_), between USF+EtOH and SF+EtOH or USF alone (Fig 3B and 3C). The levels of the ω-6 PUFA, linoleic acid (LA, C_18_H_32_O_2_), the major FA in the USF diet, were elevated in the USF+EtOH group when compared to the SF+EtOH group, although the difference was not statistically significant. Further, the levels of α-linolenic acid (ALA, C_18_H_30_O_2_, ω-3), were elevated, while the abundance of eicosapentaenoic (EPA, C_20_H_30_O_2_, ω-3) and docosahexaenoic (DHA, C_22_H_32_O_2_, ω-3) acids was low in USF+EtOH fed mice compared to SF+EtOH fed animals. However, these changes in ω-3 PUFAs were not significant due to high variability between the animals.

### Alterations in hepatic oxylipins caused by chronic-binge ethanol exposure

**Fig 4:**
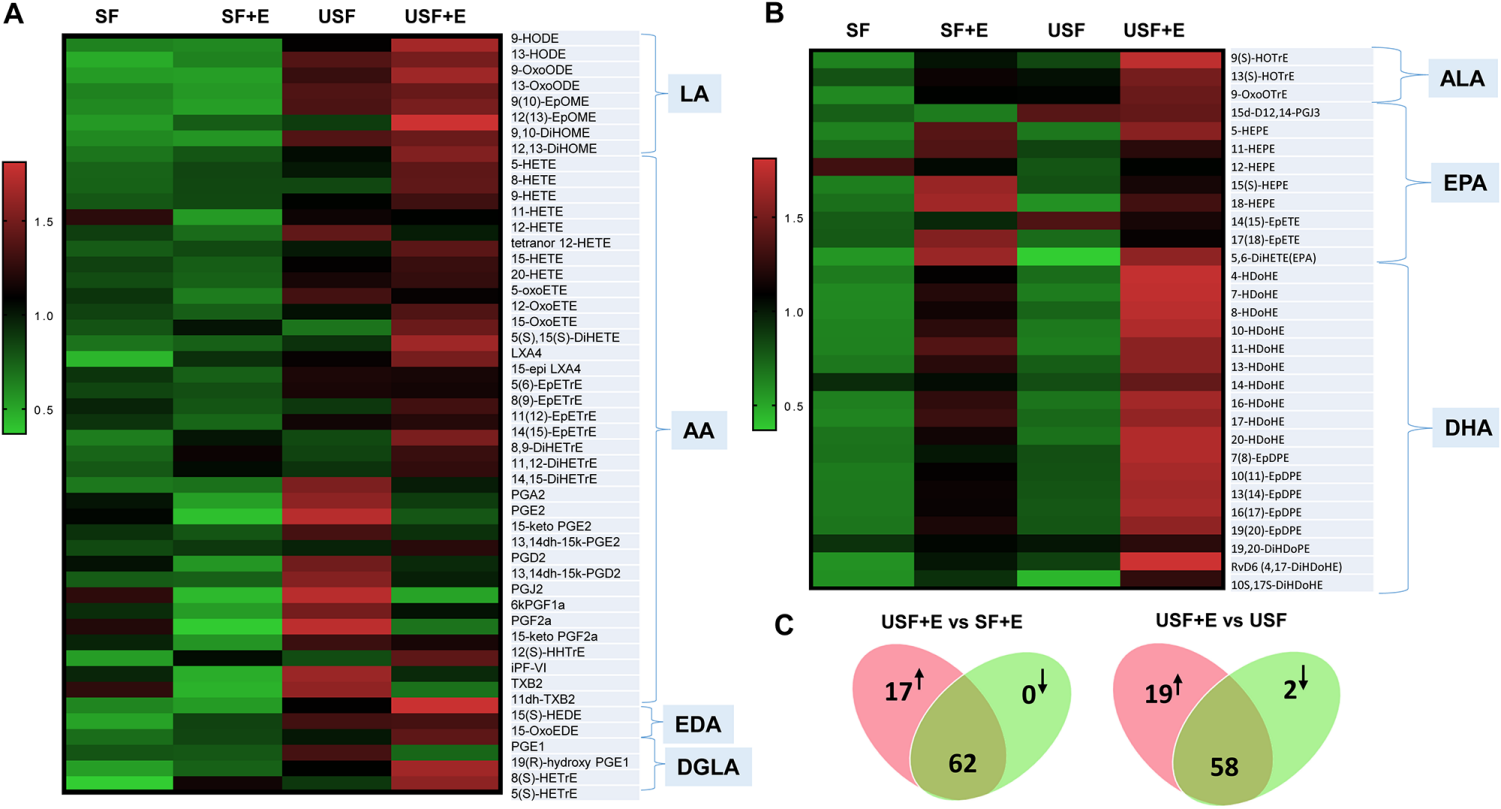
Alterations in hepatic oxylipins caused by chronic-binge ethanol exposure. A, B: Heat maps representing the overall changes in hepatic metabolites derived from ω-6 and ω-3 PUFAs occurred between experimental groups. Results are expressed as a matrix view where rows represent individual metabolites and columns represent group distribution. The intensity of each color denotes the standardized ratio between each value and the average levels of each metabolite across all samples in all groups. C: The diagram summarizing differential levels of all PUFA-derived metabolites between the indicated treatment groups. n = 6 mice per group. AA, arachidonic acid; ALA, α-linolenic acid; DHA, docosahexaenoic acid; DGLA, dihomo-γ-linolenic acid; E, ethanol; EDA, eicosadienoic acid; EPA, eicosapentaenoic acid; SF, saturated fat; USF, unsaturated fat; Individual oxylipin abbreviations see in S2 and S4 Tables.

We then performed a targeted lipidomic analysis to measure hepatic levels of bioactive lipid metabolites generated from their precursor PUFAs, generated predominantly via three major enzymatic pathways, LOX, COX and CYP/sEH. For analysis, individual metabolites were grouped according to their parent PUFAs and enzymatic origin. Heat maps (Fig 4A and 4B) reveal global changes in hepatic oxylipins derived from ω-6 and ω-3 PUFAs occurred in response to different types of dietary fat and EtOH exposure. Our major goal was to identify changes associated with severe damage (EtOH-induced steatohepatitis) compared to minimal (steatosis only) or no liver damage. Therefore, in this analysis we focused on the differences in the USF+EtOH vs SF+EtOH groups and in the USF+EtOH vs USF (pair-fed control) groups. Collectively, a total of 17 oxylipins of 79 metabolites from several lipid categories were found to be significantly different (increased) in the USF+EtOH compared to SF+EtOH group, and 21 lipid metabolites were different (19 increased and 2 decreased) between the USF+EtOH and control USF-diet fed animals (Fig 4C). A detailed oxylipin analysis for all experimental groups is provided in S4 and S6 Tables.

### Analysis of changes in hepatic oxidized lipid metabolites derived from ω-6 PUFAs

**Fig 5:**
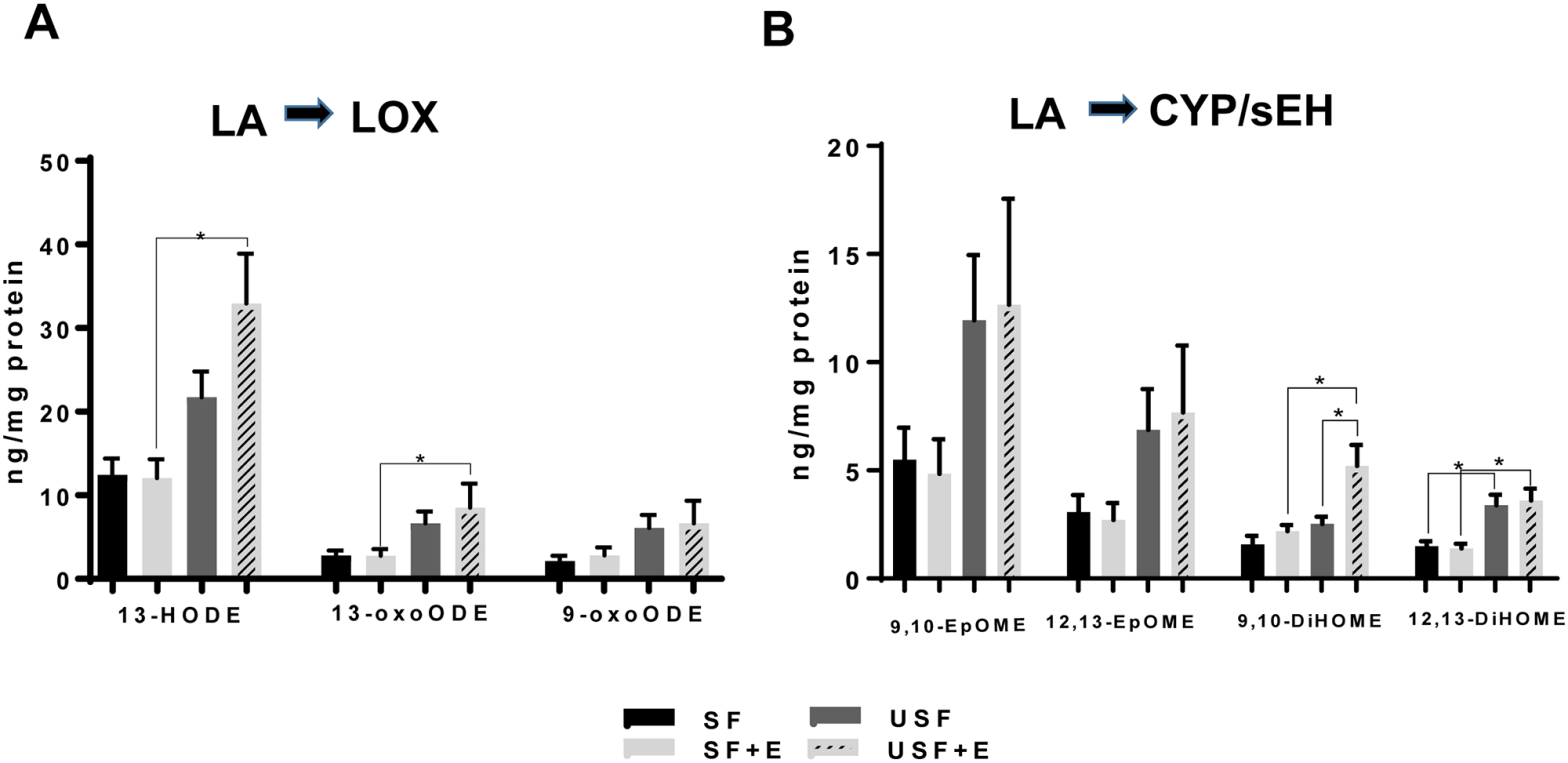
Ethanol-mediated changes in hepatic oxylipins derived from linoleic acid. A: LOX-derived hydroxy-metabolites of LA. B: Changes in epoxy- and dihydroxy-metabolites of LA derived via CYP/sEH pathway. Data are presented as means +SEM, Two way ANOVA, * *P* < 0.05, n = 6 animals per group. CYP, cytochrome-P450 epoxygenase; E, ethanol; LA, linoleic acid; LOX, lipoxygenase; SF, saturated fat; sEH, soluble epoxide hydrolase; USF, unsaturated fat. Individual oxylipin abbreviations in S2 Table.

The metabolites of LA, followed by those of AA were generally present at the higher amounts than all others (S4 Table). Specifically, 13-HODE, an LA metabolite of the LOX pathway, was the most abundant metabolite identified (Fig 5A). A trend toward higher 13-HODE concentrations was observed in the USF compared to SF group, but only reaching statistical significance in the USF+EtOH group when compared to the SF+EtOH group (32.92±5.9 vs 21.73±3.1 ng/per mg protein, respectively). 9-HODE, another oxidized LA product, was detected in less than half of samples in the SF pair-fed group and was therefore excluded from the analysis; however, it is important to note that the 9-HODE levels were significantly higher in USF+EtOH compared to SF+EtOH group (data not shown). The levels of 13 oxo-octadecadienoic acid (13-oxoODE), the downstream product of 13-HODE, were significantly increased in the USF+EtOH group when compared to the SF+EtOH group, while 9-oxoODE abundance was similar in all experimental groups (Fig 5A). CYP-generated LA metabolites, 9,10-EpOME and 12,13-EpOME, were elevated (albeit not significantly) in the USF and USF+EtOH groups when compared to the SF and SF+EtOH groups, respectively (Fig 5B). However, the levels of dihydroxy-octadecenoic acids, the diols 9,10-DiHOME and 12,13-DiHOME, formed through the action of sEH, were significantly higher in the USF+EtOH vs. SF+EtOH group (5.21±0.9 vs 2.19±0.003 ng/per mg protein and 3.61+0.56 vs 1.39±1.0 ng/per mg protein, respectively). Further, the amount of 9,10-DiHOME was 2-fold higher in USF+EtOH group relative to USF alone, and 12,13-DiHOME was significantly elevated (~2-fold) in the USF compared to SF group (Fig 4B). The ratio between corresponding diol:epoxide pairs (9,10-DIMOME:EpOME and 12,13-DIHOME:EpOME) which is an indirect measure of sEH activity, were similar between all experimental groups (S5 Table).

**Fig 6:**
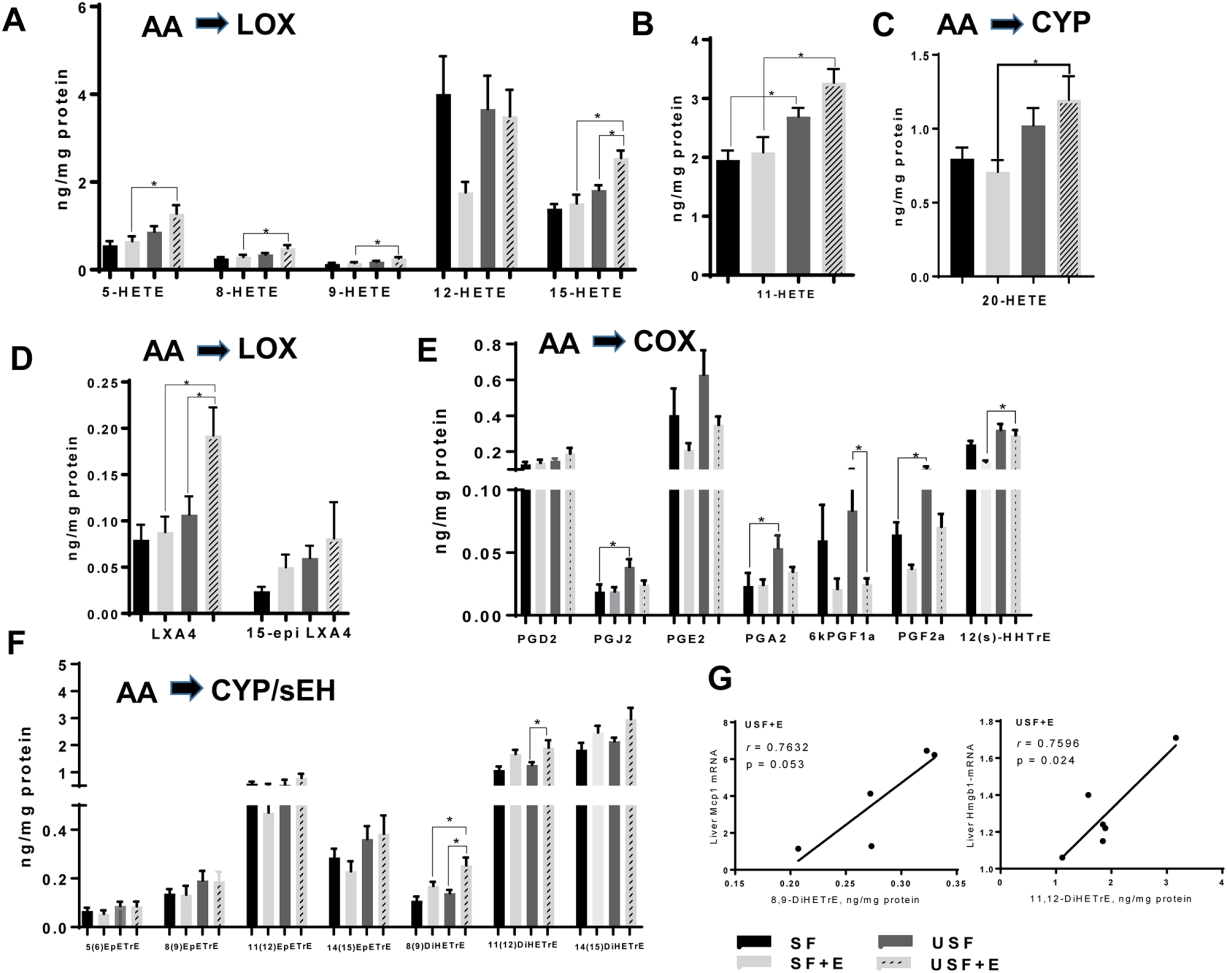
Changes in hepatic oxylipins derived from arachidonic acid. A: Changes in the profile of LOX-dependent hydroxy-metabolites of AA. B: Levels of 11-HETE. C: Levels of 20-HETE (COX pathway). D: Levels of lipoxins (LOX pathway). E: Levels of AA-derived prostaglandins (COX pathway). F: Changes in epoxy- and dihydroxy-metabolites of AA derived via CYP/sEH pathway. G: Pearson correlation analysis between AA metabolites and markers of hepatic inflammation and injury: 8,9-DiHETrE and *Mcp1* mRNA, and 11,12-DiHETrE and *Hmgb1* mRNA. Data are presented as the mean ± SEM, Two way ANOVA, * *P* < 0.05, n = 6 animals per group. AA, arachidonic acid; CYP, cytochrome-P450 epoxygenase; E, ethanol; LOX, lipoxygenase; SF, saturated fat; sEH, soluble epoxide hydrolase; USF, unsaturated fat. See oxylipin abbreviations in S2 Table.

Among AA-derived products, the HETEs (hydroxy-FAs, primarily LOX-derived oxidized products) were predominant metabolites, with the highest levels for 12-HETE (Fig 6A). Numerous pro-inflammatory HETEs, including 5-, 8-, 9-, 15-HETEs, as well as 11-HETE (non-enzymatic AA product, and a marker of oxidative stress [8]) and 20-HETE were significantly increased in the livers of mice fed USF+EtOH compared to SF+EtOH fed animals (Fig 6A, 6B and 6C). Notably, plasma levels of 5-, 8-, 9-, 11-, and 15-HETEs were also elevated in USF+EtOH compared to SF+EtOH fed animals (IK and AF, unpublished data). The levels of AA-derived anti-inflammatory, pro-resolving molecules such as the lipoxin LXA4 (LOX pathway), were significantly elevated in the USF+EtOH group when compared to the SF+EtOH as well as USF pair-fed control animals (Fig 6D). Eicosanoids, including prostaglandins and thromboxanes are the major AA products of the COX pathway. There were no differences between the USF+EtOH and SF+EtOH groups in the levels of AA-derived prostaglandins (Fig 5E), with the exception of 12(S)-hydroxy-heptadecatrienoic acid (12(S)-HHTrE, a PGH_2_ derivative), which was elevated in the USF+EtOH compared to SF+EtOH group. The levels of prostaglandins, PGJ_2_, PGA_2_, and PGF_2a_ were significantly increased in USF- compared to SF-diet fed animals; and the 6-keto-prostaglandin, F1a (6kPGF1a, a product of prostacyclin PGI_2_), was decreased in USF+EtOH vs USF group. Further, there were no differences observed in the levels of epoxy-eicosatrienoic acids (EpETrEs, the AA-derived epoxy-FAs formed via CYP pathway), including 5,6-EpETrE, 8,9-EpETrE, 11,12-EpETrE, and 14,15-EpETrE (Fig 6F). Among the dihydroxy-eicosatrienoic acids (DiHETrEs, the AA-derived dihydroxy-FAs formed via the CYP/sEH pathway), 8,9-DiHETrE was significantly higher in the USF+EtOH when compared to the SF+EtOH group (Fig 6F). Additionally, 8,9-DiHETrE as well as 11,12-DiHETrE were markedly elevated in USF+EtOH vs. USF-fed mice. Notably, the expression of these two oxylipins in the USF+EtOH group was significantly correlated with markers of liver injury and inflammation, specifically *Mcp1* and *Hmgb1* expression (Fig 6G and 6H), supporting an important role of these lipid mediators in ALD. The 14,15-DiHETrE:EpETrE ratio was elevated by EtOH, however, this increase did not reach statistical significance (S3 Table). While DiHOME:EpOME ratios (LA derivatives) were relatively small, the ratios of DiHETrE:EpETrE pairs (AA products) were markedly elevated, specifically for 11,12-DiHETrE:EpETrE (in a range of 2 to 4 fold), and for 14,15-DiHETrE:EpETrE (in a range from 6 to 12 fold with the profound effect of EtOH to increase this ratio, p<0.05), suggesting a shift toward 11,12-DiHETrE and 14,15-DiHETrE overproduction. This might be due to the increased sEH activity (substrate specific) or alterations in DiHETrEs degradation.

Among metabolites of other ω-6 PUFAs, 15S-hydroxy-eicosadienoic acid (15(S)-HEDE), a metabolite of eicosadienoic acid (EDA), was found in significantly higher levels in the livers of mice fed USF+EtOH compared to both USF and SF+EtOH groups (S4 Table). 8(S)-hydroxy-eicosatrienoic acid (8(S)-HETrE), a LOX pathway metabolite of dihomo-γ-linolenic acid (DGLA, ω-6), was significantly elevated in USF+EtOH- vs SF+EtOH-fed animals (S4 Table).

### Analysis of changes in hepatic oxidized lipid metabolites derived from ω-3 PUFAs

**Fig 7:**
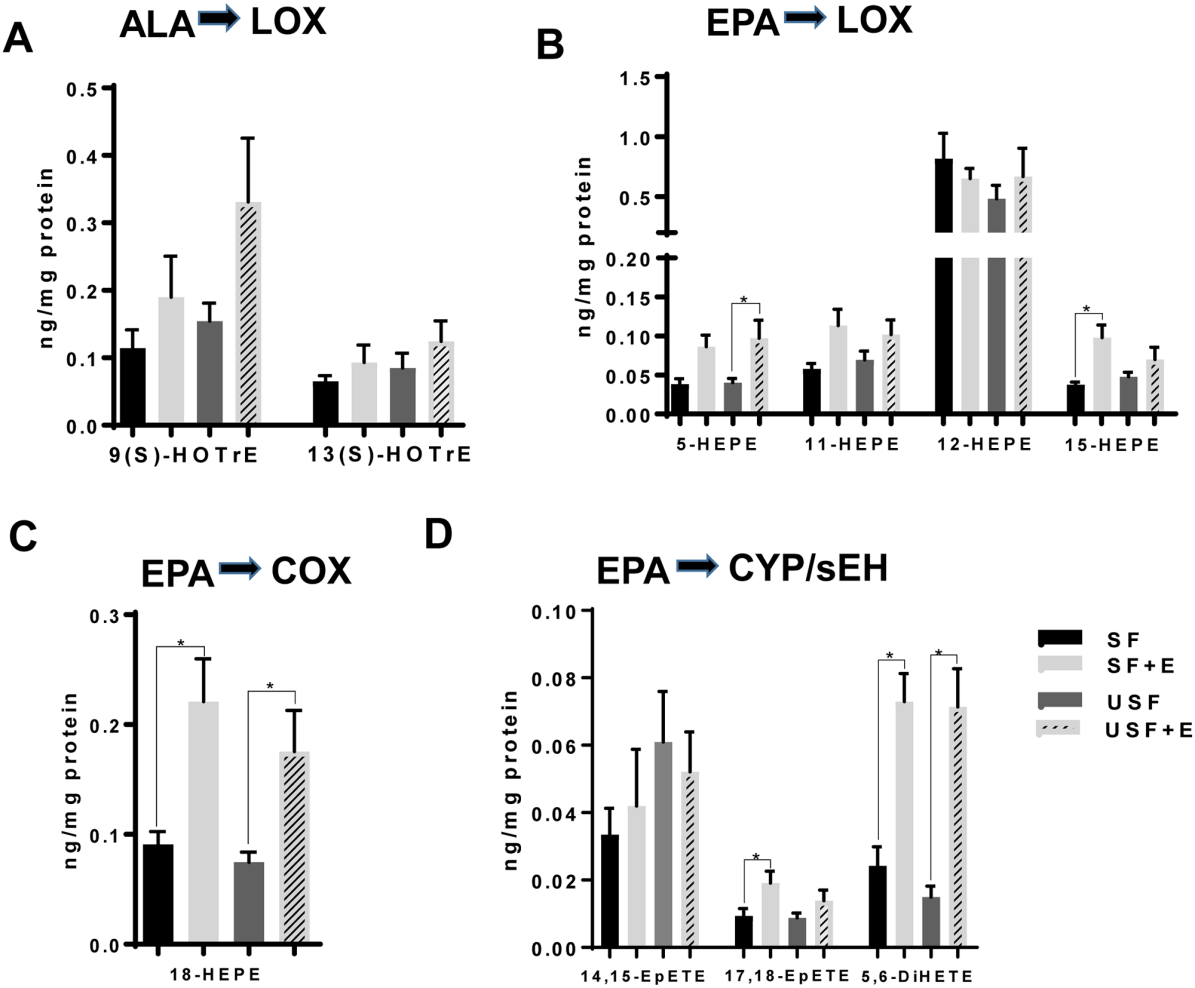
Changes in hepatic oxylipins derived from ω-3 PUFAs, linolenic and EPA. A: Levels of ALA hydroxy-metabolites of LOX pathway. B. Changes in the profile of LOX-dependent hydroxy-metabolites of EPA. C: 18-HEPE levels, COX pathway. D: Changes in epoxy- and dihydroxy-metabolites of EPA derived via CYP/sEH pathway. Data are presented as the mean ± SEM, Two way ANOVA, * *P* < 0.05, n = 6 animals per group. ALA, alpha linolenic acid; CYP, cytochrome-P450 epoxygenase; E, ethanol; EPA, eicosapentaenoic acid; LOX, lipoxygenase; SF, saturated fat; sEH, soluble epoxide hydrolase; USF, unsaturated fat. See oxylipin abbreviations in S6 Table.

Among LOX-mediated, ALA-derived hydroxy-octadecatrienoic acids (HOTrEs, products with known anti-inflammatory effects [28]), 13(S)-HOTrE and 9(S)-HOTrE were elevated (albeit not significantly) in response to EtOH exposure in both USF and SF-fed mice (Fig 7A). The levels of EPA-derived hydroxy-eicosapentaenoic acids (HEPEs, LOX-mediated metabolites), *e*.*g*., 5-, 11-, and 15S-HEPEs, were higher in the USF+EtOH compared to USF group, reaching statistical significance for 5-HEPE (Fig 7B). Interestingly, 18-HEPE, a precursor for the resolvin E series (RvE), was significantly increased in both the SF+EtOH and USF+EtOH groups compared to pair-fed controls (Fig 7C). There were no differences found in the levels of epoxy-eicosatetraenoic acids (EpETEs, EPA-products from the CYP pathway, specifically 14,15- and 17,18-EpETEs), between USF+EtOH and SF+EtOH groups (Fig 7D). However, EtOH exposure resulted in elevated 17,18-EpETE levels in both USF+EtOH and SF+EtOH groups compared to the control pair-fed animals reaching statistical significance in the SF+EtOH compared to SF control group. EpETEs are rapidly metabolized by sEH to their corresponding dihydroxy-eicosatetraenoic acids (DiHETEs). In our study, we detected only the 5,6-DiHETE metabolite, which was significantly increased by EtOH regardless the type of dietary fat (Fig 7D).

**Fig 8:**
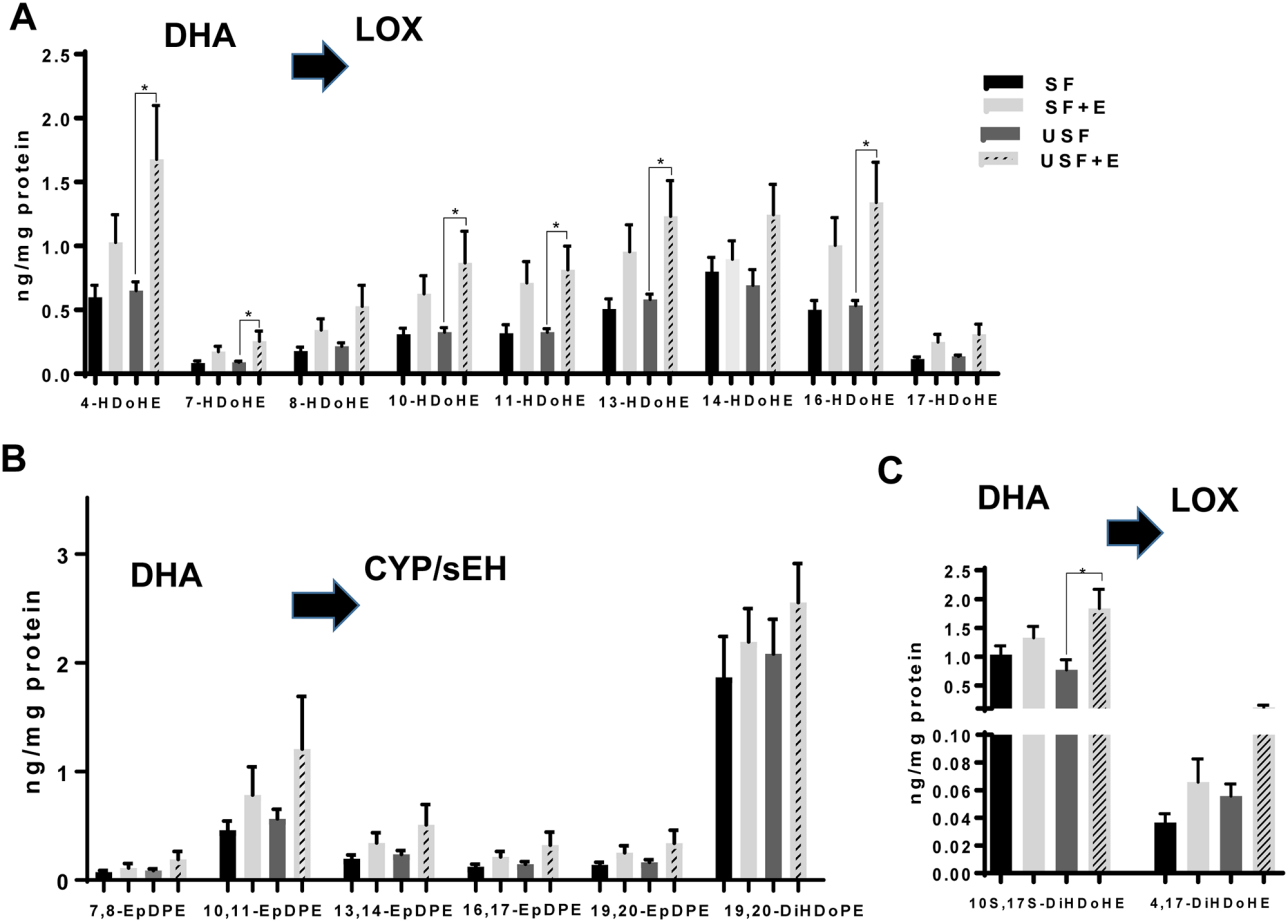
Changes in hepatic oxylipins derived from DHA. A: Levels of DHA-derived hydroxyl-metabolites of LOX pathway. D: Changes in epoxy- and dihydroxy-metabolites of DHA produced via CYP/sEH pathway. C: Pro-resolving DHA metabolites of LOX pathway. Data are presented as the mean ± SEM, Two way ANOVA, * *P* < 0.05, n = 6 animals per group. CYP, cytochrome-P450 epoxygenase; DHA, docosahexaenoic acid; E, ethanol; LOX, lipoxygenase; SF, saturated fat; sEH, soluble epoxide hydrolase; USF, unsaturated fat. See oxylipin abbreviations in S6 Table.

Interestingly, analysis of DHA metabolites revealed a significant effect of EtOH in the USF group, but not in SF-fed animals. Thus, as compared to USF diet alone, USF+EtOH fed animals had a number of significantly increased DHA-derived hydroxy-docosahexaenoic acids (HDoHE, primarily generated via LOX pathway), including 4-, 7-, 10-, 11-, 13-, and 16- HDoHEs (Fig 8A), and 20-HDoHE (CYP pathway, S4 Table). There were no significant changes in DHA-derived epoxy-docosapentaenoic acids (EpDPE, Fig 8B). Notably, several DHA metabolites related to a novel class of pro-resolving lipid mediators, such as 10S,17S-dihydroxy-docosahexaenoic acid (10S,17S-DiHDoHE, known as a protectin D1 isomer), and 4,17-DiHDoHE were increased by EtOH in the USF+EtOH fed mice (Fig 8C).

### Analysis of the expression of genes involved in hepatic oxylipin metabolism

**Fig 9:**
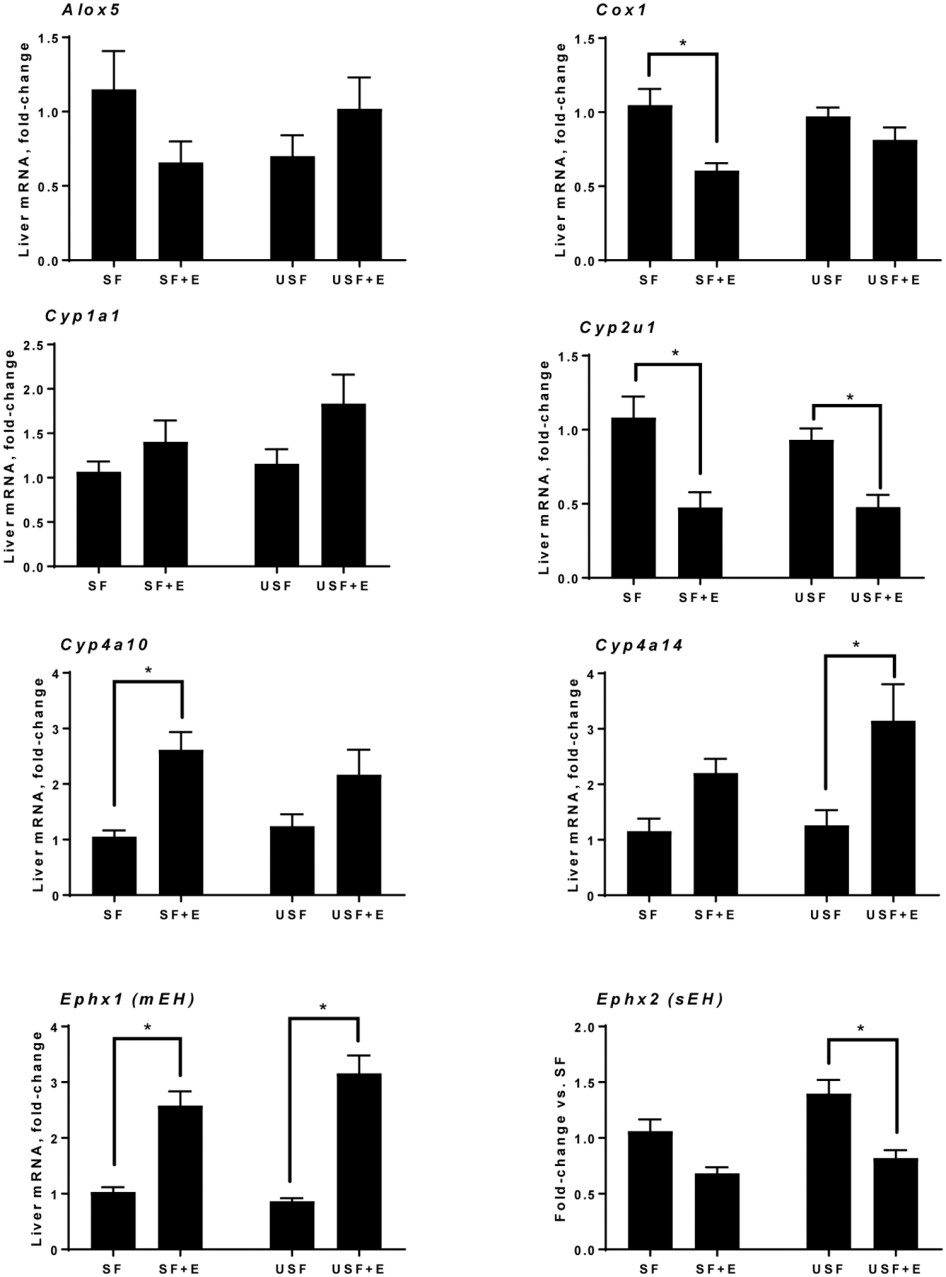
Analysis of the expression of genes involved in hepatic oxylipin production. Data are presented as the mean ± SEM vs. the SF group, two-way ANOVA, * *P* < 0.05, n = 6 animals per group.

Next, we evaluated the expression of genes from the three metabolic pathways involved in oxylipin synthesis, namely the LOX, COX and CYP/epoxide hydrolase pathways (Fig 9). Specifically, the expression of *Alox5*, a representative of the LOX pathway, was not markedly affected either by diets or EtOH. Within the COX pathway, the expression of *Cox1* was significantly reduced in the SF+EtOH but not in the USF+EtOH mice compared to pair-fed controls. Expression of the *Alox15 and* inducible COX, *Cox2*, was very low in our samples (data not shown). We evaluated the expression of four CYP pathway genes: *Cyp1a1*, *Cyp2u1*, *Cyp4a10*, and *Cyp4a14*. There was no change in the expression of *Cyp1a1*, whereas there was significant down-regulation of *Cyp2u*1 by EtOH in both SF and USF fed mice compared to pair-fed animals. *Cyp4a10*, on the other hand, was significantly increased by EtOH in the SF but not in the USF diet group compared to controls, while *Cyp4a14* was increased to a greater extent in the USF+EtOH group. Further, we analyzed the expression of *Ephx1* (microsomal epoxide hydrolase, mEH) and *Ephx2* (soluble epoxide hydrolase, sEH). The expression of *Ephx1* was increased by EtOH in both diet groups, while, the expression of *Ephx2* was decreased by EtOH, in both groups reaching statistical significance in the USF+EtOH fed group.

## Discussion

In this study, we demonstrated that the type of dietary fat modulated the severity of chronic-binge EtOH-induced liver injury, which was more advanced in mice fed USF+EtOH compared to animals fed SF+EtOH diets, and it was associated with specific pattern of bioactive ω-6 and ω-3 PUFA metabolites in the liver. Previous studies have shown that SF diet (primarily enriched in medium chain fatty acids, MCFAs) protected against hepatic steatosis and injury caused by chronic EtOH feeding in rodents [29–31]. However, in the present study, the acute binge ethanol exposure combined with chronic ethanol feeding may account for the increased hepatic fat accumulation in both SF+EtOH and USF+EtOH fed mice. Indeed, binge EtOH administration by oral gavage (single or multiple binges) in mice resulted in microvesicular fat accumulation in the liver via mechanisms involved histone deacetylase (HDAC)-mediated alterations in lipid synthesis and oxidation [32]. The differences in fat accumulation between the USF+EtOH and SF+EtOH groups (less in SF+EtOH) can be partially attributed to the metabolic properties of MCFAs, such as specific transport through the portal vein and rapid beta-oxidation in the liver [33], or to the alterations in lipid metabolism regulation. In our study, up-regulation of *Fas* and *Acc1*, genes involved in lipogenesis, likely accounts for the increased lipid accumulation in the livers of mice fed USF+EtOH compared to SF+EtOH, as the expression of *Cpt1α*, a key regulator of fatty acid oxidation, and *Fat*/*Cd36*, a gene involved in fatty acid transport, were similarly expressed (down- and up-regulated, respectively) in both USF+EtOH and SF+EtOH fed mice. Induction of lipogenic genes, including *Fas* and *Acc1*, and/or down-regulation of *Cpt1α* was previously observed in chronic, chronic-binge [34, 35], and binge [32] EtOH-induced ALD in rodents. We also found that the expression of *Scd1* was down-regulated in the USF diet group vs. the SF diet group both in pair-fed and EtOH-fed mice. Although SCD1 is known to be lipogenic, it has been previously shown that inhibition of *Scd1* expression leads to the increased hepatic triglyceride levels in rodents [36]. Therefore, lower expression of *Scd1* in the USF and USF+EtOH fed mice might be one of the contributing mechanisms to the increased liver triglycerides found in these groups. Although levels of some proteins were not altogether similar to the levels of their corresponding genes, this may be a reflection of complex, temporal changes in the expression of these proteins in our experimental model. While *Ppar-α*, a transcription factor that regulates genes involved in FFA β-oxidation, including *Cpt1a* and *Acox1* was down-regulated only in USF+EtOH, *Cpt1a* levels were reduced in both USF+EtOH and SF+EtOH mice, suggesting *Cpt1a* regulation by other mechanisms, *e*.*g*., epigenetic regulation by HDAC3 [37]. HNF4α, a transcription factor which was reduced by EtOH and dietary USF but not SF [38] might also be involved in increased hepatic fat accumulation observed in mice fed USF+EtOH. Further, up-regulation of *Srebp1*, a transcription factor implicated in the induction of genes encoding lipogenic enzymes [39, 40], in the livers of ethanol and USF but not SF diet fed mice might also contribute to the enhanced hepatic steatosis in mice fed USF+EtOH. Therefore, in our study, elevated lipid synthesis, rather than differences in fatty acid oxidation or transport, most likely contributed to the enhanced hepatic steatosis in USF+EtOH compared to SF+EtOH-fed mice.

The mechanisms of progression from alcohol-induced steatosis to steatohepatitis are not well understood. There is increasing interest in bioactive ω-6 PUFA metabolites as potential mediators of this process. Indeed, these metabolites were associated with pro-inflammatory responses and liver damage of alcoholic and non-alcoholic origin in humans and in experimental rodents [2, 3, 5–7, 16, 41, 42]. The novelty of the current study is that we performed a comprehensive analysis of hepatic oxylipins generated from ω-6 as well as ω-3 PUFAs in response to chronic-binge EtOH exposure and different types of dietary fat, USF and SF, as to their potential mechanistic role in EtOH-mediated liver injury. Using a targeted lipidomic approach, we analyzed metabolites of several ω-6 and ω-3 PUFAs, including LA- and ALA-derived octadecanoids, AA- and EPA-derived eicosanoids, DHA-derived docosanoids, as well as metabolites of EDA and DGLA generated via the LOX, COX, and CYP/sEH pathways. We identified several ω-6 PUFA and ω-3 PUFA metabolites that were differentially expressed in mice with alcoholic steatohepatitis (USF+EtOH fed mice) compared to mice with ethanol-induced steatosis (SF+EtOH fed mice) or mice without liver damage (control pair-fed animals). In line with previous observations in humans [3] and in rodent models of ALD [7, 15, 16, 43], we found EtOH-mediated increases in the LA-oxidized metabolites, 9-HODE and 13-HODE, the most abundant products of LOX pathway, in mice with marked liver injury and inflammation caused by USF+EtOH. The ability of 9-HODE to induce a pro-inflammatory response in macrophages [15] and of 13-HODE to induce oxidative stress, endoplasmic reticulum stress and apoptosis in hepatocytes, may be underlying mechanisms by which liver injury occurs in USF+EtOH-fed mice [16]. The fact that the USF experimental diet contained high levels of LA suggested that not only EtOH-mediated activation of LOX enzymes [3, 16], but also availability of LA as a substrate are critical factors for OXLAM overproduction. Further, increased parental FAs (LA in this case) might cause similar changes in their oxylipin production by a mass action effect. This in an important consideration given that LA intake in Western societies extremely high (up to 17 percent of energy [%E]), compared to historical and evolutionary norms of 2–3%E [44]. In addition, a Western diet has an imbalanced (elevated) ω-6/ω-3 PUFA ratio. The ω-6 PUFAs may compete with the ω-3 PUFAs as substrates of COX, LOX, as well as CYP enzymes, resulting in a shift towards production of largely pro-inflammatory ω-6 PUFA mediators as compared to the beneficial ω-3 PUFA products. Another abundant group of LOX-mediated products was the group of AA hydroxy-metabolites, including 5-, 8-, 9-, and 15-HETEs, which were markedly increased in USF+EtOH compared to SF+EtOH-fed mice. In addition, 11-HETE (a non-enzymatic AA product, and a marker of oxidative stress) and 20-HETE (COX pathway) were also elevated in the USF+EtOH compared to the SF+EtOH group. In general, HETEs are known for their pro-inflammatory and pro-apoptotic properties (*e*.*g*., 5-HETE increases TNF-α induced apoptosis in hepatocytes) [45], and neutrophil chemotactic activity [46] suggesting a role in promoting inflammation in USF+EtOH group. However, some HETEs have recently been recognized as anti-inflammatory mediators. For example, 15-HETE, which was elevated in USF+EtOH compared to both SF+EtOH and USF pair-fed control groups, is a PPARɣ ligand and can decrease inflammation via inhibition of nuclear factor-kB and modulate macrophage activation toward an anti-inflammatory phenotype [47, 48]. Further, 15-HETE, is a precursor to the lipoxin, LXA_4_, which was significantly increased in the USF+EtOH group [49]. Lipoxins, unlike the classical pro-inflammatory AA products, have potent anti-inflammatory and pro-resolving activities [17, 50]. In addition, 15-HETE is further oxidized to 15-oxoETE, an oxylipin with anti-inflammatory activity [51]. Therefore, our data suggest simultaneous synthesis of both pro- and anti- inflammatory eicosanoids and/or a temporal shift in lipid mediators toward an anti-inflammatory response as a part of the phenomenon termed lipid class switching [52].

The data obtained in our study point to the importance of the CYP/sEH axis in ALD. The CYP enzymes metabolize numerous PUFAs and generate a variety of different signaling lipids, including epoxy-FAs, which are rapidly hydrolyzed by sEH to their corresponding vicinal diols, dihydroxy-FAs. Alterations in epoxy- and dihydroxy-FAs have been implicated in pathogenesis of numerous pathologies, including liver disease of different origins [41, 42, 53–56]. Epoxy-FAs possess potent cytoprotective, anti-oxidant, and anti-inflammatory properties, mediate the resolution phase of inflammation [57], and can be beneficial in preventing or ameliorating the severity of liver diseases of different etiologies, *e*.*g*., NAFLD [58, 59]. In hepatocytes, epoxy-FAs (primarily AA-derived mediators, EpETrEs), improved autophagy and attenuated palmitate-induced accumulation of intracellular lipids and ER stress [18, 42]. In adipose tissue, EpETrEs inhibited macrophage recruitment and preserved the alternatively activated macrophage (M2) phenotype [60]. While the levels of epoxy-FAs in our study were similar across all precursors between all experimental groups, there were significant changes in their sEH—mediated metabolites, dihydroxy-FAs (diols). Diols were long considered to be less active than their parent epoxides, however, recent evidence demonstrated their significant biological activity [42, 61–63]. One of the important observations from our study was the increased levels of the LA dihydroxy metabolites, 9,10-DiHOME and 12,13-DiHOME, in USF+EtOH-fed mice (the group with increased hepatic neutrophil-mediated inflammation and injury) compared to USF-fed control and SF+EtOH-fed animals. The biosynthesis of these compounds begins via formation of LA epoxides, 9,10-EpOME and 12,13-EpOME, by inflammatory leucocytes such as neutrophils and macrophages [64] [65]. Both the EpOMEs and the DiHOMEs induced G-protein-mediated-chemotaxis in human neutrophils, and chemokinesis [66]. 9,10-DiHOME exerts a toxic activity toward mitochondria [67]; whether this effect would occur in hepatocytes or other cells in the liver remains to be determined. Further, DiHOMEs suppress the neutrophil respiratory burst, a critical reaction in the immune-mediated elimination of microorganisms [68]. Given that neutrophils play important roles in alcohol-induced liver damage, the significance of these LA bioactive metabolites needs to be further investigated. Little is known about the biological role of the AA-derived 8,9-DiHETrE and 11,12-DiHETrE, the other dihydroxy-FAs which were significantly increased in mice with liver injury and inflammation (USF+EtOH group); although it has been shown that DiHETrEs may activate PPARα and 14,15-dihydroxyeicosatrienoic acid increased the expression of *Cpt1a*, a PPARα-responsive gene, in HepG2 cells [62]. The functional significance of the DiHETrE increase in ALD needs further investigation. Overall, the fact that PUFA epoxy- metabolites were not altered by EtOH administration, while increases in several dihydroxy-mediators, derivatives of sEH pathway, were associated with alcoholic steatohepatitis points to novel and as yet undiscovered roles for sEH and bioactive molecules derived via this pathway in the pathogenesis of ALD. CYP enzymes and sEH are highly expressed in the liver [69], inhibition of sEH is beneficial in liver pathologies of different origins (*e*.*g*., high fat diet-mediated NAFLD [54], and CCl_4_-induced hepatic fibrosis [53]). Thus, alterations in the CYP/sEH axis may play important roles in the pathogenesis of ALD and may therefore be targets that have potential therapeutic significance as they do for several other diseases [56]. More detailed studies are needed to determine the role of the different PUFA epoxides and diols in the pathophysiology of ALD and the therapeutic potential of sEH inhibition either alone or in combination with dietary PUFA modulation.

EPA and DHA are precursors to numerous metabolites, many of which (e.g., resolvins, protectins, and maresins) possess anti-inflammatory and pro-resolving properties [17, 70, 71]. There were several hydroxy- metabolites of EPA whose concentrations were significantly changed, including 5-, 15-, and 18-HEPEs. 5-HEPE (LOX product), which was increased in the USF+EtOH group *vs*. USF alone, is known to reduce the inflammatory response of macrophages [72]. The marked elevation of hepatic 18-HEPE (COX product) by EtOH in both SF and USF dietary groups is of particular interest because this EPA-derived metabolite is the precursor to the resolvins of the E-series (RvE), further suggesting that inflammation and resolution of inflammation in our experimental model of ALD are tightly connected processes. Several HDoHEs, DHA hydoxy-metabolites generated via LOX pathway, were significantly increased in USF+EtOH compared to USF-fed control group. The function of HDoHEs themselves has not been well elucidated, but some are precursors to pro-resolving lipid mediators [17]; for example, 17-HDoHE is the precursor to resolvins of the D-series (RvD), and 14-HDoHE is the precursor to maresin 1. In addition, 10S,17S-DiHDoHE (protectin DX [73]) and 4,17-DiHDoHE (precursor for RvD6) were also increased by EtOH in USF-fed group, suggesting enhanced resolution of the inflammatory response in these mice. The fact that elevated levels of pro-resolving molecules in the USF+EtOH group were associated with marked liver inflammation and injury suggest that the resolution of inflammation likely was initiated as a natural/adaptive response to inflammation; however, the pro-resolving molecules might not be able to exert their beneficial effects due to compromised resolvin/receptor interactions. Limited information is available regarding the effects of EtOH on the structure and function of FPR2/ALX (formyl peptide receptor type 2 activation by lipoxin A4) and GPR32 (G protein-coupled receptor 32), well recognized RvD1 receptors, and ChemR23 (chemerin receptor 23) and BLT1 (leukotriene B4 receptor 1), RvE1 receptors. The effects of EtOH on downstream resolvin/receptor signaling mechanisms are also not known. Furthermore, protectins and resolvins may be potential treatments for ALD, but more research into this area is needed.

Oxylipins are formed primarily via three major metabolic pathways namely LOX, COX, and CYP/EH. In some cases, the same oxylipins can be produced from more than one pathway, thus making attempts to correlate the changes in the expression of specific genes with the production of specific oxylipins challenging. Of interest in our study was the effects of EtOH and diet on the expression of genes involved in oxylipin production. We did not identify any diet-specific effects on the expression of selected genes from either LOX, COX, or CYP/EH pathways. EtOH, however, led to a down-regulation of *Cox1* (one of the main enzymes in prostaglandin synthesis) in the SF+EtOH group, which was associated with an attenuated inflammatory response observed in these mice compared to animals fed USF+EtOH. Further, mice fed EtOH exhibited differential hepatic expression of several *Cyp450* genes, including *Cyp2u1*, *Cyp4a10*, and *Cyp4a14*, with both up- and down-regulation in the SF-EtOH and USF-EtOH fed animals. Interestingly, *Cyp4a14* knockout mice have attenuated high fat diet-induced hepatic steatosis whereas *Cyp4a14* overexpression promoted fat accumulation [74]. It is possible that the increased *Cyp4a14* expression in the USF+EtOH group contributed to the elevated hepatic triglycerides observed in this group, suggesting that alterations in oxylipin metabolism may contribute to the pathological changes in the liver, including steatosis and inflammation.

**Fig 10:**
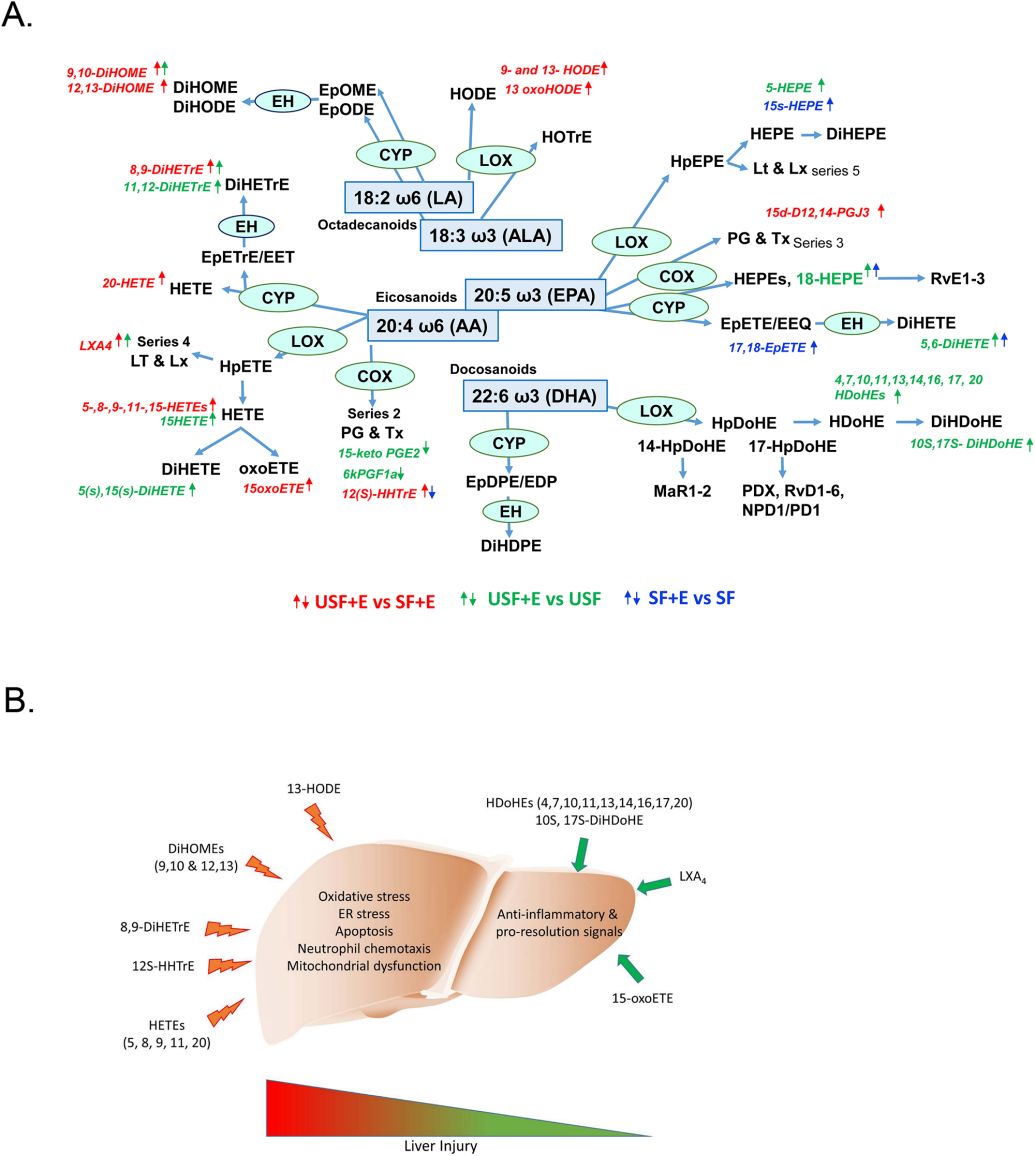
EtOH-mediated alterations in liver oxylipins. A: Summary of changes in hepatic ω-6 and ω-3 PUFA-derived oxylipins in response to EtOH exposure. B: A working model wherein multiple ω-6-derived oxylipins (to the left) facilitate liver injury via distinct mechanisms. The deleterious effects of these oxylipins outweigh the benefits of anti-inflammatory and pro-resolving bioactive lipid mediators (to the right), and tips the balance towards liver damage. Abbreviations are listed in S4 and S6 Tables.

In summary, we identified diet-specific changes in the hepatic ω-6 and ω-3 PUFA-derived oxylipins in response to chronic-binge EtOH administration in mice (Fig 10A). The significant USF+EtOH-mediated increase in harmful, pro-inflammatory metabolites of ω-6 PUFAs may underlie the more severe liver pathology observed in these mice. We propose a working model wherein multiple ω-6-derived oxylipins facilitate liver injury via distinct mechanisms, including inflammation, oxidative stress, ER stress, and mitochondrial dysfunction (Fig 10B). The deleterious effects of these oxylipins outweigh the benefits of anti-inflammatory and pro-resolving bioactive lipid mediators, and tips the balance towards liver damage. Although oxylipins have been demonstrated to function via multiple pathways, further research is required to elucidate the exact mechanisms by which they exert their effects during ALD pathogenesis. In conclusion, the potentially hepatotoxic oxylipins identified in this study may be new biomarkers and novel therapeutic targets for ALD.

## Supporting information

S1 Methods (DOCX)

S1 Table Primer sequences for qPCR assays. (DOCX)

S2 Table Antibodies used for western blotting. (DOCX)

S3 Table Hepatic fatty acid levels in mice exposed to chronic-bing EtOH administration. (DOCX)

S4 Table Hepatic levels of ω-6 PUFA metabolites. (DOCX)

S5 Table The sEH product/substrate ratio of selected diol-epoxide pairs. (DOCX)

S6 Table Hepatic levels of ω-3 PUFA metabolites. (DOCX)

## Abbreviations

AA: arachidonic acid
Acc1: acetyl-CoA carboxylase 1
ALA: α-linolenic acid
ALD: alcoholic liver disease
ALT: aminotransferase
BLT1: leukotriene B4 receptor 1
CAE: chloroacetate esterase
ChemR23: chemerin receptor 23
COXs: cyclooxygenases
*Cpt1α*: carnitine palmitoyltransferase 1A
CYPs: cytochrome-P450 epoxygenases
DGLA: dihomo-γ-linolenic acid
DHA: docosahexaenoic acid
DiHDoHE: dihydroxy-docosahexaenoic acid
DiHDoPE: dihydroxy-docosapentaenoic acid
DiHETE: dihydroxy-eicosatetraenoic acid
DiHETrE: dihydroxy-eicosatrienoic acid
DiHOME: dihydroxy-octadecenoic acid
EDA: eicosadienoic acid
EPA: eicosapentaenoic acid
EpDPE: epoxy-docosapentaenoic acid
EpETE: epoxy-eicosatetraenoic acid
EpETrE = EET: epoxy-eicosatrienoic acid
EpOME: epoxy-octadecenoic acid
EtOH: ethanol
Fasn: fatty acid synthase
*Fat/Cd36*: fatty acid translocase/cluster of differentiation 36
FPR2/ALX: formyl peptide receptor type 2 activation by lipoxin A4
GPR32: G protein-coupled receptor 32
HDAC: histone deacetylase
HDoHE: hydroxy-docosahexaenoic acid
HEDE: hydroxy-eicosadienoic acid
HEPE: hydroxy-eicosapentaenoic acid
HETE: hydroxy-eicosatetraenoic acid
HETrE: hydroxy-eicosatrienoic acid
HHTrE: hydroxy-heptadecatrienoic acid
HODE: hydroxy-octadecadienoic acid
Il-1β: interleukin-1 beta
iP: isoprostane
LA: linoleic acid
LOXs: lypoxygenases
LX: lipoxin
MCFAs: medium chain fatty acids
Mcp1: monocyte chemotactic protein-1
MCT: medium chain triglycerides
NAFLD: nonalcoholic fatty liver disease
OxoEDE: oxo-eicosadienoic acid
OxoETE: oxo-eicosatetraenoic acid
OxoODE: oxo-octadecadienoic acid
OxoOTrE: oxo-octadecatrienoic acid
PG: prostaglandins
*Ppar-α*: peroxisome proliferator-activated receptor α
*Scd1*: stearoyl CoA desaturase
sEH: soluble epoxide hydrolase
SF: saturated fat
*Srebp1*: sterol regulatory element-binding protein 1
TGs: triglycerides
*Tnf-α*: tumor necrosis factor alpha
TX: thromboxane
USF: unsaturated fat
