# Supplementary material for: Ethanol and unsaturated dietary fat induce unique patterns of hepatic ω-6 and ω-3 PUFA oxylipins in a mouse model of alcoholic liver disease
Source: PLoS One. 2018 Sep 26;13(9):e0204119. doi: 10.1371/journal.pone.0204119 (PMC6157879; doi:10.1371/journal.pone.0204119)
Supplement: S1 Methods — (DOCX) [file pone.0204119.s001.docx]

**Supplemental Methods**

**Hepatic FFA analysis**

**Sample Preparation**: The sample group information was first removed and all samples were then processed in a random order to avoid systemic bias. A sample of liver tissue was weighed and then homogenized for 15 min in water at a ratio of 100 mg liver tissue/mL water. 400 µL of an 80% methanol solution was added to 100 µL sample. The mixture was vortexed for 2 min and then placed on ice for 10 min. After another 2 min vortex, the sample was centrifuged at 4°C, 11200 rpm for 20 min. After transferring 400 µL of supernatant into a glass vial, it was first dried in a Speedvac (Thermo Fisher) to remove methanol, followed by freeze drying to remove water. For sample preparation for GC×GC-TOF MS, each metabolite extract was dissolved in 30 µL of pyridine with 20 mg/mL methoxyamine hydrochloride and vigorously vortex-mixed for 1 min. Methoxymation was carried out by sonicating the solution for 20 min followed by 1 h incubation at 60°C. Derivatization was conducted by adding 20 µL of N-methyl-N-(trimethylsilyl) trifluoroacetamide (MSTFA). The solution was incubated at 60 °C for another 1 h. The stock solutions were then transferred to GC sample vials for analysis. The methoxymation and derivatization were carried out just before GC×GC-TOF MS analysis. Pooled samples were prepared simultaneously by mixing 50-100 µL sample supernatants and then conducting methoxymation and derivatization. Pooled samples were analyzed on GC×GC-TOF MS after analysis of every 8 biological samples.

**GC×GC-TOF MS Analysis**: A LECO Pegasus GC×GC-TOF MS instrument was coupled to an Agilent 6890 gas chromatography and a Gerstel MPS2 autosampler (GERSTEL Inc., Linthicum, MD), featuring a LECO two-stage cryogenic modulator and secondary oven. The primary column was a 60 m × 0.25 mm 1dc × 0.25 µm 1dp DB-5 ms GC capillary column (phenyl arylene polymer virtually equivalent to (5%-phenyl)-methylpolysiloxane). The secondary GC column 1 m × 0.25 mm 1dc × 0.25 µm 1df, DB-17 ms ((50% phenyl)-methylpolysiloxane) was placed inside the secondary GC oven following the thermal modulator. Both columns were obtained from Agilent Technologies (Agilent Technologies J&W, Santa Clara, CA). The helium carrier gas (99.999% purity) flow rate was set to 2.0 mL/min at a corrected constant flow via pressure ramps. The inlet temperature was set to 280 °C. The primary column temperature was programmed with an initial temperature of 60 °C for 0.5 min, then ramped at 5°C/min to 270°C, and maintained for 13 min. the secondary column temperature program was set to an initial temperature of 70 °C for 0.5 min and then also ramped at the same temperature gradient employed in the first column to 280 °C, accordingly. The thermal modulator was set to + 15°C relative to the primary oven, and a modulation time of Pm = 2 s was used. The mass range was set as 29-800 m/z with an acquisition rate of 200 mass spectra per second. The ion source chamber was set at 230 °C with the transfer line temperature of 280 °C, and the detector voltage was set 1400 V with electron energy of 70 eV. The acceleration voltage was turned on after a solvent delay of 544 s, the split ratio was set at 5:1. The internal standard signals in each chromatogram were used for normalization for recovery as well as relative quantitation of each analyte. Arbitrary units (peak areas) were used to analyze the abundance of free fatty acids in the liver tissue.
