# Supplementary material for: Ethanol and unsaturated dietary fat induce unique patterns of hepatic ω-6 and ω-3 PUFA oxylipins in a mouse model of alcoholic liver disease
Source: PLoS One. 2018 Sep 26;13(9):e0204119. doi: 10.1371/journal.pone.0204119 (PMC6157879; doi:10.1371/journal.pone.0204119)
Supplement: S1 Table — (DOCX) [file pone.0204119.s002.docx]

**S1 TABLE. Primer Sequences for qPCR assays**

| **Gene** | **Forward** | **Reverse** |
| --- | --- | --- |
| *18s* | 5′-CTCAACACGGGAAACCTCAC-3′ | 5′-CGCTCCACCAACTAAGAACG-3′ |
| *Acc1* | 5’-CTTCCTGACAAACGAGTCTGG-3’ | 5’-CTGCCGAAACATCTCTGGGA-3’ |
| *Acox1* | 5’-TTCCTGCCCACCTTGCTTCA-3’ | 5’-AGCCTCGAAGATGAGTTCCATGA-3’ |
| *Alox 5* | 5’-GTCCGAGTACCTGACGGTGG-3’ | 5’-GCACCAGTCATACTGGCCGA-3’ |
| *Cd36* | 5’-ATGGGCTGTGATCGGAACTG-3’ | 5’-GTCTTCCCAATAAGCATGTCTCC-3’ |
| *Chrebp1* | 5’-ATGACCCCTCACTCAGGGAATA-3’ | 5’-GATCCAAGGGTCCAGAGCAG-3’ |
| *Cox1* | 5’-GTGCCCTCACCAGTCAATCC-3’ | 5’-GGTACAGTTGGGGCCTGAGT -3’ |
| *Cpt1a* | 5’-GCTGCACTCCTGGAAGAAGA-3’ | 5’-GGAGGGGTCCACTTTGGTAT-3’ |
| *Cyp1a1* | 5’-GGTTAACCATGACCGGGAACT-3’ | 5’-TGCCCAAACCAAAGAGAGTGA-3’ |
| *Cyp2u1* | 5’-TCGCCATTCCTCACATGACCT-3’ | 5’-CGATGAGGACAGAAGTCGTCTG-3’ |
| *Cyp4a10* | 5’-ACCCTGCATAGTCTCTTTCTACCTG-3’ | 5-GTGCATGACACTGGGAACTTT-3’ |
| *Cyp4a14* | 5’-GCTCACGAGCACACAGATGG-3’ | 5’-GGCCTTCTGCAGCTCTTCCT-3’ |
| *Ephx1* | 5’-GGAGACCTTACCACTTGAAGATG-3’ | 5’-GCCCGGAACCTATCTATCCTCT-3’ |
| *Ephx2* | 5’-ACCACTCATGGATGAAAGCTACA-3’ | 5’-TCAGGTAGATTGGCTCCACAG-3’ |
| *Fasn* | 5’-CAGTGGGTGGACTCTCTGAAG | 5’-GACAGCAGGATACACCGAATC-3’ |
| *Hmgb1* | 5’-GGCGAGCATCCTGGCTTATC-3’ | 5’-GGCTGCTTGTCATCTGCTG-3’ |
| *Il1-β* | 5′-TTCATCTTTGAAGAAGAGCCCAT-3′ | 5′-TCGGAGCCTGTAGTGCAGTT-3′ |
| *Mcp1* | 5′-GGCTCAGCCAGATGCAGT-3′ | 5′-TGAGCTTGGTGACAAAAACTACAG-3′ |
| *Pparα* | 5’-AGAGCCCCATCTGTCCTCTC-3’ | 5’-ACTGGTAGTCTGCAAAACCAAA-3’ |
| *Scd1* | 5’-TTCTTGCGATACACTCTGGTGC-3’ | 5’-CGGGATTGAATGTTCTTGTCGT-3’ |
| *Srebp1c* | 5’-GGAGCCATGGATTGCACATT-3’ | 5’-GCTTCCAGAGAGGAGGCCAG-3’ |
| *Tnf-α* | 5′-GTGATCGGTCCCCAAAGG-3′ | 5’-GGTGGTTTGCTACGACGTG-3’ |
| *Xbp1* | 5’-GGTCTGCTGAGTCCGCAGCAGG-3’ | 5’-AGGCTTGGTGTATACATGG-3’ |
