# Supplementary material for: Ethanol and unsaturated dietary fat induce unique patterns of hepatic ω-6 and ω-3 PUFA oxylipins in a mouse model of alcoholic liver disease
Source: PLoS One. 2018 Sep 26;13(9):e0204119. doi: 10.1371/journal.pone.0204119 (PMC6157879; doi:10.1371/journal.pone.0204119)
Supplement: S2 Table — (DOCX) [file pone.0204119.s003.docx]

**S2 TABLE. Antibodies used in this study**

| **Protein** | **Vendor** | **Antibody Registry No.** | **Dilution** |
| --- | --- | --- | --- |
| ACC1 | Abcam | AB 867475 | 1:1000 |
| β-ACTIN | Santa Cruz Biotech. | AB 2223230 | 1:1000 |
| CPT1a | Proteintech | AB 2084676 | 1:1000 |
| FASN | Santa Cruz Biotech. | AB 2101095 | 1:1000 |
| GAPDH | Santa Cruz | AB 10167668 | 1:1000 |
| LSD1 | Cell Signaling Tech. | AB 2070132 | 1:5000 |
| PPAR-α | Santa Cruz Biotech. | AB 2165737 | 1:1000 |
| SCD1 | Abcam | AB 445179 | 1:1000 |
