# Supplementary material for: Ethanol and unsaturated dietary fat induce unique patterns of hepatic ω-6 and ω-3 PUFA oxylipins in a mouse model of alcoholic liver disease
Source: PLoS One. 2018 Sep 26;13(9):e0204119. doi: 10.1371/journal.pone.0204119 (PMC6157879; doi:10.1371/journal.pone.0204119)
Supplement: S3 Table — (DOCX) [file pone.0204119.s004.docx]

**S3 TABLE. Hepatic fatty acid levels in mice exposed to chronic-binge ethanol administration**

| **Fatty Acid** | **SF** | **SF+EtOH** | **USF** | **USF+EtOH** | **Two-Way ANOVA, *P* values** | | |
| --- | --- | --- | --- | --- | --- | --- | --- |
|  |  |  |  |  | ***P_1_*** | ***P_2_*** | ***P_3_*** |
| Decanoic acid, C_10_H_20_O_2_ | 7.89+1.81 | 9.14+1.23 | 4.88+0.86 | 5.09+0.75 | 0.6099 | 0.0181 | 0.7145 |
| Dodecanoic acid, C_12_H_24_O_2_ | 7.02+0.93 | 15.63+3.40 ^a^ | 5.41+0.70 | 5.41+0.46 ^b^ | 0.0280 | 0.0036 | 0.0283 |
| Palmitic acid, C_16_H_32_O_2_ | 112.4+10.0 | 96.66+6.18 | 111.5+8.69 | 121.5+7.98 | 0.7488 | 0.1851 | 0.1547 |
| Stearic acid, C_18_H_36_O_2_ | 130.5+12.05 | 123.1+10.43 | 134.7+12.5 | 129.6+10.89 | 0.5948 | 0.6509 | 0.9233 |
| Oleic acid, C_18_H_34_O_2_ | 110.8+12.19 | 129.1+12.1 | 105.8+15.95 | 118.9+12.11 | 0.2498 | 0.5742 | 0.8450 |
| Linoleic acid, C_18_H_32_O_2_ | 258.1+17.01 | 263.9+19.5 | 288.1+21.12 | 310.8+14.74 | 0.4484 | 0.0471 | 0.6538 |
| Arachidonic acid, C_20_H_32_O_2_ | 22.84+2.43 | 28.69+3.21 | 27.36+1.81 | 25.58+2.05 | 0.4936 | 0.6943 | 0.1991 |
| α-Linolenic acid, C_18_H_30_O_2_ | 1.85+0.91 | 1.92+0.33 | 2.81+1.18 | 3.32+0.35 | 0.7088 | 0.1290 | 0.7730 |
| Eicosapentaenoic acid, C_20_H_30_O_2_ | 4.92+1.58 | 9.46+1.78 | 3.32+0.85 | 4.81+0.52 | 0.0520 | 0.0446 | 0.3143 |
| Docosahexaenoic acid, C_22_H_32_O_2_ | 32.6+6.81 | 63.33+8.59 ^a^ | 25.46+3.26 | 41.47+2.88 | 0.0011 | 0.0325 | 0.2634 |

Data are presented as peak area x 10^-4^, mean+SEM, n=6-10. ^a^ SF vs SF+EtOH; ^b^ SF+EtOH vs USF+EtOH. Two-way ANOVA was performed to assess the individual contribution of EtOH or diet, and their interaction. *P_1_* is the *P* value of ethanol factor, *P_2_* is the *P* value of a diet factor, *P_3_* is the *P* value of the interaction between diet and ethanol. EtOH, ethanol, SF, saturated fat; USF, unsaturated fat.
