# Supplementary material for: Ethanol and unsaturated dietary fat induce unique patterns of hepatic ω-6 and ω-3 PUFA oxylipins in a mouse model of alcoholic liver disease
Source: PLoS One. 2018 Sep 26;13(9):e0204119. doi: 10.1371/journal.pone.0204119 (PMC6157879; doi:10.1371/journal.pone.0204119)
Supplement: S4 Table — (DOCX) [file pone.0204119.s005.docx]

**S4 Table. Hepatic levels of ω-6 PUFA metabolites**

| **Lipid Mediators** | **SF** | **SF+EtOH** | **USF** | **USF+EtOH** | **Two-Way Anova, *P* values** | | |
| --- | --- | --- | --- | --- | --- | --- | --- |
|  |  |  |  |  | ***P_1_*** | ***P_2_*** | ***P_3_*** |
| **Linoleic Acid** | | | | | | | |
| 13-HODE | 12.454 ± 1.954 | 12.064 ± 2.26 | 21.735 ± 3.065 | 32.925 ± 5.987 ^a^ | 0.1578 | 0.0006 | 0.1313 |
| 9-OxoODE | 2.159 ± 0.595 | 2.796 ± 0.987 | 6.103 ± 1.528 | 6.666 ± 2.685 | 0.7197 | 0.0280 | 0.9823 |
| 13-OxoODE | 2.818 ± 0.596 | 2.760 ± 0.792 | 6.648 ± 1.427 | 8.530 ± 2.852 ^a^ | 0.5912 | 0.0094 | 0.5677 |
| 9,10-EpOME | 5.498 ± 1.479 | 4.838 ± 1.602 | 11.939 ± 3.015 | 12.661 ± 4.897 | 0.9921 | 0.0311 | 0.8245 |
| 12,13-EpOME | 3.077 ± 0.778 | 2.705 ± 0.778 | 6.867 ± 1.894 | 7.681 ± 3.094 | 0.9083 | 0.0316 | 0.4579 |
| 9,10-DiHOME | 1.583 ± 0.395 | 2.195 ± 0.286 | 2.525 ± 0.327 | 5.214 ± 0.957 ^ab^ | 0.0081 | 0.0021 | 0.0792 |
| 12,13-DiHOME | 1.498 ± 0.220 | 1.388 ± 0.227 | 3.391 ± 0.489 ^c^ | 3.607 ± 0.556 ^a^ | 0.8959 | 0.0001 | 0.6898 |
| **Arachidonic Acid** | | | | | | | |
| 5-HETE | 0.558 ± 0.095 | 0.651 ± 0.110 | 0.866 ± 0.125 | 1.278 ± 0.197 ^a^ | 0.0811 | 0.0028 | 0.2603 |
| 8-HETE | 0.257 ± 0.030 | 0.294 ± 0.047 | 0.349 ± 0.032 | 0.492 ± 0.068 ^a^ | 0.0698 | 0.0057 | 0.2708 |
| 9-HETE | 0.134 ± 0.021 | 0.151 ± 0.023 | 0.181 ± 0.017 | 0.256 ± 0.032 ^a^ | 0.0826 | 0.0067 | 0.2602 |
| 11-HETE | 1.995 ± 0.162 | 2.084 ± 0.260 | 2.692 ± 0.149 ^c^ | 3.269 ± 0.235 ^a^ | 0.1042 | 0.0002 | 0.2937 |
| 12-HETE | 4.006 ± 0.864 | 1.763 ± 0.241 | 3.662 ± 0.762 | 3.496 ± 0.607 | 0.0838 | 0.3063 | 0.1322 |
| tetranor 12-HETE | 0.052 ± 0.010 | 0.043 ± 0.008 | 0.086 ± 0.014 | 0.059 ± 0.021 | 0.2280 | 0.0945 | 0.5676 |
| 15-HETE | 1.393 ± 0.107 | 1.511 ± 0.199 | 1.817 ± 0.112 | 2.549 ± 0.168 ^ab^ | 0.0108 | 0.0001 | 0.0561 |
| 20-HETE | 0.797 ± 0.076 | 0.708 ± 0.081 | 1.021 ± 0.119 | 1.195 ± 0.161 ^a^ | 0.7157 | 0.0055 | 0.2635 |
| 5-oxoETE | 0.258 ± 0.045 | 0.236 ± 0.041 | 0.368 ± 0.079 | 0.399 ± 0.081 | 0.9448 | 0.0457 | 0.6786 |
| 12-OxoETE | 0.028 ± 0.003 | 0.020 ± 0.005 | 0.041 ± 0.004 | 0.034 ± 0.005 | 0.1304 | 0.0085 | 0.9003 |
| 15-OxoETE | 0.026 ± 0.004 | 0.023 ± 0.003 | 0.031 ± 0.003 | 0.041 ± 0.005 ^a^ | 0.3552 | 0.0044 | 0.0832 |
| 5(S),15(S)-DiHETE | 0.026 ± 0.006 | 0.034 ± 0.004 | 0.022 ± 0.002 | 0.048 ± 0.008 ^b^ | 0.0143 | 0.1072 | 0.1185 |
| LXA4 | 0.080 ± 0.016 | 0.088 ± 0.016 | 0.107 ± 0.020 | 0.192 ± 0.030 ^ab^ | 0.0424 | 0.0063 | 0.0887 |
| 15-epi LXA4 | 0.024 ± 0.005 | 0.050 ± 0.014 | 0.060 ± 0.013 | 0.081 ± 0.039 | 0.2984 | 0.1414 | 0.9193 |
| 5,6-EpETrE=EET | 0.066. ± 0.013 | 0.055 ± 0.014 | 0.087 ± 0.017 | 0.085 ± 0.019 | 0.6885 | 0.1240 | 0.7576 |
| 8,9-EpETrE=EET | 0.138 ± 0.018 | 0.133 ± 0.037 | 0.192 ± 0.036 | 0.188 ± 0.039 | 0.9028 | 0.1279 | 0.9816 |
| 11,12-EpETrE=EET | 0.576 ± 0.078 | 0.471 ± 0.095 | 0.534 ± 0.189 | 0.786 ± 0.156 | 0.6067 | 0.3434 | 0.2208 |
| 14,15-EpETrE=EET | 0.286 ± 0.036 | 0.230 ± 0.041 | 0.362 ± 0.053 | 0.383 ± 0.076 | 0.7486 | 0.0450 | 0.4743 |
| 8,9-DiHETrE | 0.108 ± 0.017 | 0.169 ± 0.017 | 0.139 ± 0.014 | 0.253 ± 0.033 ^ab^ | 0.0006 | 0.0151 | 0.2305 |
| 11,12-DiHETrE | 1.084 ± 0.125 | 1.675 ± 0.154 | 1.264 ± 0.107 | 1.908 ± 0.278 ^b^ | 0.0025 | 0.2616 | 0.8859 |
| 14,15-DiHETrE | 1.830 ± 0.256 | 2.468 ± 0.250 | 2.145 ± 0.138 | 2.961 ± 0.425 | 0.0195 | 0.1739 | 0.7580 |
| PGA2 | 0.023 ± 0.011 | 0.024 ± 0.004 | 0.053 ± 0.012 ^c^ | 0.035 ± 0.004 | 0.2119 | 0.0073 | 0.0762 |
| PGE2 | 0.403 ± 0.149 | 0.210 ± 0.037 | 0.628 ± 0.137 | 0.349 ± 0.047 | 0.0361 | 0.0998 | 0.6885 |
| 15-keto PGE2 | 0.173 ± 0.054 | 0.067 ± 0.010 | 0.281 ± 0.059 | 0.127 ± 0.018 | 0.0052 | 0.0555 | 0.5704 |
| 13,14-dihydro-15-keto-PGE2 | 0.039 ± 0.013 | 0.034 ± 0.013 | 0.057 ± 0.010 | 0.040 ± 0.007 | 0.3099 | 0.3099 | 0.5880 |
| PGD2 | 0.128 ± 0.014 | 0.137 ± 0.018 | 0.149 ± 0.011 | 0.189 ± 0.032 | 0.0612 | 0.1527 | 0.3212 |
| 13,14-dihydo-15-keto-PGD2 | 0.166 ± 0.046 | 0.091 ± 0.010 | 0.240 ± 0.043 | 0.153 ± 0.019 | 0.0225 | 0.0529 | 0.8653 |
| PGJ2 | 0.019 ± 0.006 | 0.019 ± 0.003 | 0.039 ± 0.006 ^c^ | 0.024 ± 0.003 | 0.1598 | 0.0156 | 0.1505 |
| 6-keto-PGF1a | 0.060 ± 0.028 | 0.021 ± 0.008 | 0.083 ± 0.019 | 0.025 ± 0.005 ^b^ | 0.0117 | 0.2293 | 0.4314 |
| PGF2a | 0.064 ± 0.010 | 0.037 ± 0.003 | 0.103 ± 0.013 ^c^ | 0.071 ± 0.010 | 0.0062 | 0.0015 | 0.7767 |
| 15-keto-PGF2a | 0.058 ± 0.023 | 0.018 ± 0.004 | 0.084 ± 0.020 | 0.033 ± 0.006 | 0.0084 | 0.2089 | 0.7084 |
| 12(S)-HHTrE | 0.240 ± 0.021 | 0.141 ± 0.009 ^d^ | 0.321 ± 0.034 | 0.291 ± 0.030 ^a^ | 0.0184 | 0.0002 | 0.1872 |
| iPF-VI | 0.006 ± 0.001 | 0.012 ± 0.001 | 0.009 ± 0.001 | 0.016 ± 0.002 ^b^ | 0.0023 | 0.4831 | 0.9009 |
| TXB2 | 0.194 ± 0.061 | 0.097 ± 0.01 | 0.331 ± 0.053 | 0.191 ± 0.017 | 0.0100 | 0.0119 | 0.6209 |
| 11dh-TXB2 | 0.018 ± 0.006 | 0.006 ± 0.001 | 0.022 ± 0.006 | 0.010 ± 0.002 | 0.0426 | 0.1233 | 0.4249 |
| **Eicosadienoic Acid** | | | | | | | |
| 15(S)-HEDE | 0.025 ± 0.003 | 0.022 ± 0.003 | 0.044 ± 0.004 | 0.072 ± 0.011 ^ab^ | 0.0686 | <0.0001 | 0.0239 |
| 15-OxoEDE | 0.014 ± 0.003 | 0.024 ± 0.011 | 0.037 ± 0.013 | 0.037 ± 0.012 | 0.6311 | 0.1003 | 0.6655 |
| **Dihomo-γ-Linolenic Acid** | | | | | | | |
| PGE1 | 0.023 ± 0.005 | 0.028 ± 0.002 | 0.033 ± 0.002 | 0.047 ± 0.010 | 0.0790 | 0.0752 | 0.2778 |
| 19(R)-hydroxy PGE1 | 0.002 ± 0.000 | 0.002 ± 0.000 | 0.004 ± 0.001 | 0.002 ± 0.000 ^b^ | 0.0791 | 0.1543 | 0.0791 |
| 8(S)-HETrE | 0.098 ± 0.016 | 0.138 ± 0.035 | 0.200 ± 0.031 | 0.305 ± 0.076 ^a^ | 0.1247 | 0.0077 | 0.4746 |
| 5(S)-HETrE | 0.045 ± 0.018 | 0.140 ± 0.040 | 0.109 ± 0.019 | 0.193 ± 0.077 | 0.0615 | 0.2082 | 0.9060 |

Data are presented as ng analyte/mg protein (mean+SEM, n=4-6), Two-way ANOVA was performed to assess the contribution of the ethanol, diet, and their interactions. *P_1_* is the *P* value of ethanol factor, *P_2_* is the *P* value of a diet factor, *P_3_* is the *P* value of the interaction between the diet and ethanol. Values with different superscripts differ significantly (P < 0.05). ^a^ SF+EtOH vs USF+EtOH; ^b^ USF+EtOH vs USF; ^c^ SF vs USF; ^d^ SF vs SF+EtOH. DiHETE, dihydroxy-eicosatetraenoic acid; DiHETrE, dihydroxy-eicosatrienoic acid; DiHOME, dihydroxy-octadecenoic acid; EpETrE=EET, epoxy-eicosatrienoic acid; EpOME, epoxy-octadecenoic acid; EtOH, ethanol; HEDE, hydroxy-eicosadienoic acid; HETE, hydroxy-eicosatetraenoic acid; HETrE, hydroxy-eicosatrienoic acid; HHTrE, hydroxy-heptadecatrienoic acid; HODE, hydroxy-octadecadienoic acid; iP, isoprostane; LX, lipoxin; OxoEDE, oxo-eicosadienoic acid; OxoETE, oxo-eicosatetraenoic acid; OxoODE, oxo-octadecadienoic acid; PG, prostaglandins; SF, saturated fat; TX, thromboxane; USF, unsaturated fat.
