# Supplementary material for: Ethanol and unsaturated dietary fat induce unique patterns of hepatic ω-6 and ω-3 PUFA oxylipins in a mouse model of alcoholic liver disease
Source: PLoS One. 2018 Sep 26;13(9):e0204119. doi: 10.1371/journal.pone.0204119 (PMC6157879; doi:10.1371/journal.pone.0204119)
Supplement: S5 Table — (DOCX) [file pone.0204119.s006.docx]

**S5 Table. The sEH product/substrate ratio of selected diol-epoxide pairs**

| **Parameters** | **SF** | **SF+EtOH** | **USF** | **USF+EtOH** | **Two-Way ANOVA, *P* values** | | |
| --- | --- | --- | --- | --- | --- | --- | --- |
|  |  |  |  |  | ***P_1_*** | ***P_2_*** | ***P_3_*** |
| 9,10-DiHOME:EpOME | 0.32+0.06 | 0.55+0.09 | 0.24+0.04 | 0.52+0.11 | 0.0066 | 0.5192 | 0.7864 |
| 12,13-DiHOME:EpOME | 0.57+0.10 | 0.57+0.07 | 0.57+0.07 | 0.61+0.09 | 0.8403 | 0.8594 | 0.7895 |
| 8,9-DiHETrE:EpETrE | 0.82+0.14 | 1.61+0.23 | 0.77+0.14 | 1.59+0.34 | 0.0041 | 0.8978 | 0.9607 |
| 11,12-DiHETrE:EpETrE | 2.00+0.30 | 4.28+0.61 | 3.78+0.89 | 2.98+0.79 | 0.3062 | 0.7375 | 0.0401 |
| 14,15-DiHETrE:EpETrE | 6.98+1.45 | 12.02+1.95 | 6.31+0.70 | 9.21+2.29 | 0.0313 | 0.3235 | 0.5407 |

Data are presented as ratio of sEH product/substrate, mean+SEM, n=4-6. Two-way ANOVA was performed to assess the contribution of the ethanol, diet, and their interactions. *P_1_* is the *P* value of ethanol factor, *P_2_* is the *P* value of a diet factor, *P_3_* is the *P* value of the interaction between the diet and ethanol. DiHETrE, dihydroxy-eicosatrienoic acid; DiHOME, dihydroxy-octadecenoic acid; EpETrE, epoxy-eicosatrienoic acid; EpOME, epoxy-octadecenoic acid; EtOH, ethanol, SF, saturated fat; USF, unsaturated fat
