# Supplementary material for: Ethanol and unsaturated dietary fat induce unique patterns of hepatic ω-6 and ω-3 PUFA oxylipins in a mouse model of alcoholic liver disease
Source: PLoS One. 2018 Sep 26;13(9):e0204119. doi: 10.1371/journal.pone.0204119 (PMC6157879; doi:10.1371/journal.pone.0204119)
Supplement: S6 Table — (DOCX) [file pone.0204119.s007.docx]

**S6 Table. Hepatic Levels of ω-3 PUFA Metabolites**

| **Lipid Metabolite** | **SF** | **SF + EtOH** | **USF** | **USF + EtOH** | **Two-Way Anova, *P* values** | | |
| --- | --- | --- | --- | --- | --- | --- | --- |
|  |  |  |  |  | ***P_1_*** | ***P_2_*** | ***P_3_*** |
| **α-Linolenic Acid** | | | | | | | |
| 9(S)-HOTrE | 0.114 ± 0.027 | 0.190 ± 0.061 | 0.154 ± 0.027 | 0.331 ± 0.094 | 0.0462 | 0.1417 | 0.4023 |
| 13(S)-HOTrE | 0.065 ± 0.009 | 0.093 ± 0.026 | 0.085 ± 0.022 | 0.124 ± 0.030 | 0.1597 | 0.2837 | 0.8070 |
| 9-OxoOTrE | 0.044 ± 0.008 | 0.080 ± 0.031 | 0.079 ± 0.025 | 0.108 ± 0.043 | 0.2811 | 0.2957 | 0.9092 |
| **Eicosapentaenoic Acid** | | | | | | | |
| 15d-D12,14-PGJ3 | 0.740 ± 0.239 | 0.635 ± 0.094 | 1.399 ± 0.241 | 1.440 ± 0.258 ^a^ | 0.8846 | 0.0031 | 0.7412 |
| 5-HEPE | 0.038 ± 0.007 | 0.086 ± 0.015 | 0.040 ± 0.005 | 0.097 ± 0.023 ^b^ | 0.0017 | 0.6485 | 0.7593 |
| 11-HEPE | 0.058 ± 0.007 | 0.113 ± 0.021 | 0.069 ± 0.011 | 0.102 ± 0.019 | 0.0171 | 0.9963 | 0.4934 |
| 12-HEPE | 0.817 ± 0.211 | 0.652 ± 0.083 | 0.481 ± 0.113 | 0.667 ± 0.237 | 0.9541 | 0.3655 | 0.3238 |
| 15(S)-HEPE | 0.037 ± 0.003 | 0.098 ± 0.017 ^d^ | 0.048 ± 0.006 | 0.070 ± 0.016 | 0.0026 | 0.4608 | 0.1285 |
| 18-HEPE | 0.091 ± 0.012 | 0.221 ± 0.039 ^d^ | 0.074 ± 0.009 | 0.176 ± 0.037 ^b^ | 0.0005 | 0.2832 | 0.6096 |
| 14,15-EpETE | 0.034 ± 0.008 | 0.042 ± 0.017 | 0.061 ± 0.015 | 0.052 ± 0.012 | 0.9899 | 0.1643 | 0.5136 |
| 17,18-EpETE | 0.009 ± 0.002 | 0.019 ± 0.003 ^d^ | 0.009 ± 0.001 | 0.014 ± 0.003 | 0.0161 | 0.3103 | 0.4044 |
| 5,6-DiHETE | 0.024 ± 0.006 | 0.073 ± 0.008 ^d^ | 0.015 ± 0.003 | 0.071 ± 0.011 ^b^ | <0.0001 | 0.5002 | 0.6340 |
| **Docosahexaenoic Acid** | | | | | | | |
| 4-HDoHE | 0.598 ± 0.095 | 1.027 ± 0.217 | 0.651 ± 0.068 | 1.677 ± 0.421 ^b^ | 0.0074 | 0.1659 | 0.2358 |
| 7-HDoHE | 0.085 ± 0.017 | 0.174 ± 0.043 | 0.091 ± 0.009 | 0.255 ± 0.078 ^b^ | 0.0116 | 0.3454 | 0.4219 |
| 8-HDoHE | 0.179 ± 0.030 | 0.343 ± 0.088 | 0.217 ± 0.026 | 0.527 ± 0.165 | 0.0217 | 0.2605 | 0.4528 |
| 10-HDoHE | 0.309 ± 0.048 | 0.626 ± 0.141 | 0.326 ± 0.034 | 0.868 ± 0.249 ^b^ | 0.0082 | 0.3861 | 0.4511 |
| 11-HDoHE | 0.317 ± 0.068 | 0.711 ± 0.168 | 0.326 ± 0.027 | 0.814 ± 0.185 ^b^ | 0.0030 | 0.6714 | 0.7214 |
| 13-HDoHE | 0.508 ± 0.077 | 0.954 ± 0.212 | 0.582 ± 0.042 | 1.232 ± 0.280 ^b^ | 0.0066 | 0.3429 | 0.5793 |
| 14-HDoHE | 0.799 ± 0.112 | 0.896 ± 0.145 | 0.693 ± 0.122 | 1.244 ± 0.238 | 0.0593 | 0.4661 | 0.1762 |
| 16-HDoHE | 0.501 ± 0.073 | 1.005 ± 0.217 | 0.534 ± 0.040 | 1.341 ± 0.314 ^b^ | 0.0031 | 0.3572 | 0.4487 |
| 17-HDoHE | 0.116 ± 0.015 | 0.248 ± 0.060 | 0.136 ± 0.011 | 0.309 ± 0.080 | 0.0072 | 0.4418 | 0.6889 |
| 20-HDoHE | 0.719 ± 0.098 | 1.235 ± 0.239 | 0.728 ± 0.068 | 1.857 ± 0.362 ^b^ | 0.0016 | 0.1763 | 0.1872 |
| 7,8-EpDPE | 0.075 ± 0.013 | 0.111 ± 0.043 | 0.089 ± 0.013 | 0.191 ± 0.071 | 0.1203 | 0.2858 | 0.4439 |
| 10,11-EpDPE | 0.459 ± 0.086 | 0.784 ± 0.259 | 0.565 ± 0.088 | 1.206 ± 0.486 | 0.1025 | 0.3612 | 0.5829 |
| 13,14-EpDPE | 0.198 ± 0.034 | 0.342 ± 0.095 | 0.238 ± 0.034 | 0.508 ± 0.187 | 0.0680 | 0.3494 | 0.5662 |
| 16,17-EpDPE | 0.124 ± 0.022 | 0.213 ± 0.052 | 0.146 ± 0.023 | 0.321 ± 0.120 | 0.0645 | 0.3444 | 0.5327 |
| 19,20-EpDPE | 0.140 ± 0.025 | 0.251 ± 0.066 | 0.165 ± 0.021 | 0.340 ± 0.119 | 0.0546 | 0.4245 | 0.6500 |
| 19,20-DiHDoPE | 1.866 ± 0.375 | 2.192 ± 0.307 | 2.081 ± 0.317 | 2.553 ± 0.361 | 0.2559 | 0.4087 | 0.8321 |
| 4,17-DiHDoHE RvD6 | 0.037 ± 0.007 | 0.066 ± 0.017 | 0.056 ± 0.009 | 0.120 ± 0.040 | 0.0491 | 0.1169 | 0.4455 |
| 10S,17S-DiHDoHE | 1.035 ± 0.153 | 1.330 ± 0.197 | 0.774 ± 0.172 | 1.837 ± 0.335 ^b^ | 0.0142 | 0.6214 | 0.1359 |

Data are presented as ng analyte/mg protein (mean+SEM, n=4-6), Two-way ANOVA was performed to assess the contribution of the ethanol, diet, and their interactions. *P_1_* is the *P* value of ethanol factor, *P_2_* is the *P* value of a diet factor, *P_3_* is the *P* value of the interaction between the diet and ethanol. Values with different superscripts differ significantly (P < 0.05). ^a^ SF+EtOH vs USF+EtOH ^b^ USF+EtOH vs USF ^c^ SF vs USF ^d^ SF vs SF+EtOH. DiHDoHE, dihydroxy-docosahexaenoic acid; DiHDoPE, dihydroxy-docosapentaenoic acid; DiHETE, dihydroxy-eicosatetraenoic acid; EpDPE, epoxy-docosapentaenoic acid; EpETE, epoxy-eicosatetraenoic acid; EtOH, ethanol; HDoHE, hydroxy-docosahexaenoic acid; HEPE, hydroxy-eicosapentaenoic acid; HOTrE, hydroxy-octadecatrienoic acid; OxoOTrE, oxo-octadecatrienoic acid; PG, prostaglandins; SF, saturated fat; USF, unsaturated fat.
